# A missense variant in *SHARPIN* mediates Alzheimer’s disease-specific brain damages

**DOI:** 10.1038/s41398-021-01680-5

**Published:** 2021-11-16

**Authors:** Jun Young Park, Dongsoo Lee, Jang Jae Lee, Jungsoo Gim, Tamil Iniyan Gunasekaran, Kyu Yeong Choi, Sarang Kang, Ah Ra Do, Jinyeon Jo, Juhong Park, Kyungtaek Park, Donghe Li, Sanghun Lee, Hoowon Kim, Immanuel Dhanasingh, Suparna Ghosh, Seula Keum, Jee Hye Choi, Gyun Jee Song, Lee Sael, Sangmyung Rhee, Simon Lovestone, Eunae Kim, Seung Hwan Moon, Byeong C. Kim, SangYun Kim, Andrew J. Saykin, Kwangsik Nho, Sung Haeng Lee, Lindsay A. Farrer, Gyungah R. Jun, Sungho Won, Kun Ho Lee, for the Alzheimer’s Disease Neuroimaging Initiative

**Affiliations:** 1 Gwangju Alzheimer’s & Related Dementia cohort research center, Chosun University, Gwangju, Korea; 2 Department of Public Health Sciences, Graduate School of Public Health, Seoul National University, Seoul, Korea; 3 Neurozen Inc., Seoul, Korea; 4 Department of Biomedical Science, Chosun University, Gwangju, Korea; 5 Department of Life Science, Chosun University, Gwangju, Korea; 6 Interdisciplinary Program in Bioinformatics, Seoul National University, Seoul, Korea; 7 Department of Medicine (Biomedical Genetics), Boston University School of Medicine, Boston, MA USA; 8 Department of medical consilience, Graduate school, Dankook university, Cheonan, Korea; 9 Department of Neurology, Chosun University Hospital, Gwangju, Korea; 10 Department of Cellular and Molecular Medicine, Chosun University School of Medicine, Gwangju, Korea; 11 Department of Life Science, Chung-Ang University, Seoul, Korea; 12 Department of Medical Science, College of Medicine, Catholic Kwandong University, Gangneung, Korea; 13 Department of Software and Computer Engineering, Ajou University, Suwon-si, Korea; 14 Department of Psychiatry, University of Oxford, Oxford, UK; 15 College of Pharmacy, Chosun University, Gwangju, Korea; 16 Department of Nuclear Medicine, Samsung Medical Center, Seoul, Korea; 17 Department of Neurology, Chonnam National University Medical School, Gwangju, Korea; 18 Department of Neurology, Seoul National University College of Medicine and Clinical Neuroscience Center, Seoul National University Bundang Hospital, Seongnam-si, Gyeonggi-do, Korea; 19 Indiana Alzheimer’s Disease Research Center and Center for Neuroimaging, Department of Radiology and Imaging Sciences, Indiana University School of Medicine, Indianapolis, IN USA; 20 Department of Ophthalmology, Boston University School of Medicine, Boston, MA USA; 21 Department of Biostatistics, Boston University School of Public Health, Boston, MA USA; 22 Institute of Health and Environment, Seoul National University, Seoul, Korea; 23 RexSoft Corps, Seoul, Korea; 24 Korea Brain Research Institute, Daegu, Korea

**Keywords:** Biomarkers, Genetics

## Abstract

Established genetic risk factors for Alzheimer’s disease (AD) account for only a portion of AD heritability. The aim of this study was to identify novel associations between genetic variants and AD-specific brain atrophy. We conducted genome-wide association studies for brain magnetic resonance imaging measures of hippocampal volume and entorhinal cortical thickness in 2643 Koreans meeting the clinical criteria for AD (*n* = 209), mild cognitive impairment (*n* = 1449) or normal cognition (*n* = 985). A missense variant, rs77359862 (R274W), in the SHANK-associated RH Domain Interactor (*SHARPIN*) gene was associated with entorhinal cortical thickness (*p* = 5.0 × 10^−9^) and hippocampal volume (*p* = 5.1 × 10^−12^). It revealed an increased risk of developing AD in the mediation analyses. This variant was also associated with amyloid-β accumulation (*p* = 0.03) and measures of memory (*p* = 1.0 × 10^−4^) and executive function (*p* = 0.04). We also found significant association of other *SHARPIN* variants with hippocampal volume in the Alzheimer’s Disease Neuroimaging Initiative (rs3417062, *p* = 4.1 × 10^−6^) and AddNeuroMed (rs138412600, *p* = 5.9 × 10^−5^) cohorts. Further, molecular dynamics simulations and co-immunoprecipitation indicated that the variant significantly reduced the binding of linear ubiquitination assembly complex proteins, SHPARIN and HOIL-1 Interacting Protein (HOIP), altering the downstream NF-κB signaling pathway. These findings suggest that SHARPIN plays an important role in the pathogenesis of AD.

## Introduction

Alzheimer’s disease (AD), the most common type of dementia, is a progressive disorder that causes cognitive dysfunction and memory loss. Major risk factors for AD include age, family history, and lifestyle [1]. AD has a strong genetic component with an estimated 58–79% of its liability explained by genetic factors [2]. The *APOE* ɛ4 allele accounts for a large portion of the heritability [3–6]. Genome-wide association studies (GWAS) have identified more than 30 independent loci [7, 8], yet, more than half of the phenotypic variance still remains unexplained [5]. Detection of additional AD risk loci can be enhanced through studies of diverse populations [9–11], demonstrated by GWAS focused on African Americans [12], Hispanics [13, 14], Japanese [15, 16], Chinese [17], and transethnic approaches [18]. Studies of diverse populations can leverage allele frequency differences that often result in variable strengths in association signals with particular variants and associations that are population-specific. Furthermore, effects of disease susceptibility loci (DSL) may be modulated by environmental risk factors that differ in exposure across populations.

Some of the genetic architecture of AD is likely too difficult to uncover by GWAS with samples as large as 100,000 subjects because of the mechanistic complexity underlying the disease [7]. Dissecting AD into biologically simpler disease-related outcomes (i.e., endophenotypes) has identified many significant genetic associations with measures of cognitive performance, structural brain atrophy (quantified by magnetic resonance imaging (sMRI)), and AD proteins in CSF or brain tissue [19–24]. Here, we report findings from a GWAS of several AD-related sMRI traits in a Korean sample including individuals with AD, mild cognitive impairment (MCI), and cognitively normal (CN) functioning. We found a genome-wide significant association of a missense variant in *SHARPIN* with measures of hippocampal atrophy and cortical thickness. Subsequent analysis revealed that this variant is also significantly associated with amyloid-beta (Aβ) levels in the brain, measured by positron emission tomography (PET) scan and confirmed by assessments of cognitive function and memory. Furthermore, we demonstrated experimentally that SHARPIN functionally affects NF-kB signaling in the nervous system, suggesting plays a role in the pathophysiology of AD.

## Materials and methods

### Genome-wide association study participants

The study sample included 5570 subjects who have enrolled in the Gwangju Alzheimer’s & Related Dementia (GARD) cohort registry at Chosun University in Gwangju, Korea. At baseline, there were 2030 CN, 2184 MCI, and 1356 AD subjects. The clinical definition of each group is provided in supplementary Text 1. A subset of 629 CN and 247 MCI subjects had at least one follow-up exam between 2010 and 2020 (mean follow-up interval = 28.8 months). After follow-up, 53 CN and 21 MCI subjects were re-classified as MCI and AD, respectively, resulting in a final sample of 1927 CN, 2216 MCI, and 1377 AD subjects.

The study protocol was approved by the Institutional Review Board of Chosun University Hospital, Korea (CHOSUN 2013–12–018–070). All volunteers or authorized guardians for cognitively impaired individuals gave written informed consent before participation.

### Association analysis methods

Genotype data were preprocessed by the standard downstream quality control and imputation (Supplementary Text 2 and Supplementary Figs. S1 and S2). MRI traits (Supplementary Text 3 and Supplementary Table S1) were transformed by inverse normal transformation and GWAS were conducted for each trait using PLINK [25], ONETOOL [26], and linear regression models including imputed single nucleotide polymorphisms (SNP) genotype and covariates for age, sex, *APOE* genotype, a term for the log-transformed measure of intracranial volume (ICV), and the first three principal components (PCs) to adjust for population stratification. PC analysis was performed accounting for a genetic relationship matrix using EIGENSOFT [27]. *APOE* genotype was coded as a class variable with ε3/ε3 set as the reference and five dummy variables for ε2/ε2, ε2/ε3, ε2/ε4, ε3/ε4, and ε4/ε4. A total of 3930,740 SNPs with minor allele frequency (>0.01) were tested. The genome-wide significance (GWS) threshold was set as *p* < 5.0 × 10^−8^. We used LocusZoom [28] to generate regional plots and R software v.3.6 (R Development Core Team, Vienna, Austria) to create QQ, Manhattan, and regional plots. Follow-up analyses were performed in the ADNI [29] (*n* = 1566) and AddNeuroMed datasets [30] (*n* = 288) to replicate or extend GWS findings using similar models like those employed in the GWAS.

### Gene-based association analyses with rare variants

Gene-based analyses for hippocampal volume (HV) and entorhinal thickness were performed using the MAGMA software tool [31] and models that included the same covariates as those described in tests of individual variants. These analyses included 9,784,321 functional SNPs with MAF < 0.01 that were annotated using the SNP2GENE function implemented in the FUMA program [32]. The significance threshold was set at *p* < 2.6 × 10^−6^ to correct for 19,231 gene-based tests.

### Mediation analyses

The *SHARPIN* SNP showing GWS association with MRI traits was further evaluated in a sample CN and AD subjects (*n* = 985 and *n* = 209, respectively) to determine whether its influence on AD risk was mediated through a particular MRI trait. Mediation models were evaluated using linear regression with AD as the outcome, SNP as the predictor, and the MRI trait variables as mediators. Models also included sex, age, three PCs, and log-transformed ICV (logICV) as covariates. Mediation analyses were conducted using the PROCESS macro [33] implemented in SPSS by selecting four and 10,000 bias-corrected bootstrap samples.

### Statistical methods for testing the association between PET imaging measures of Aβ accumulation and cognitive performance

Accumulation of Aβ in the brain was measured via PET in 1377 subjects (162 AD, 587 MCI, and 628 CN subjects), using a dedicated Discovery ST PET-CT scanner (General Electric Medical Systems, Milwaukee, WI, USA). PET images were obtained from subjects 90–100 min after IV injection of a mean dose of 303 MBq 20% florbetaben, a fluorin-18-labeled stilbene derivative with the trade name of NeuraCeq [34]. Preprocessing of Aβ-PET images was performed using previously described data [35]. Visual assessment of transaxial PET images was performed by a trained reader (B. Kim), using a gray scale. Each brain region (frontal cortex, lateral temporal cortex, parietal cortex, and posterior cingulate cortex/precuneus) was visually assessed and scored according to the brain amyloid plaque load (BAPL) scoring system for each PET scan. BAPL scores of 1 were classified as Aβ-negative PET scans, while BAPL scores of 2 and 3 were classified as Aβ-positive PET scans. Among 1377 subjects, 418 had a positive BAPL and 959 had a negative BAPL (Supplemental Table S2). We employed a logistic regression model to evaluate the association of the GWS *SHARPIN* missense variant with the derived binary BAPL variable adjusted for age and sex.

Association of this SNP with five domains of cognitive performance (attention, frontal/executive function, language, memory, and visuospatial skills) assessed by the Seoul Neuropsychological Screening Battery (SNSB) [36] was tested using linear regression models including age and sex as covariates. The SNSB cognitive data were available for all 2643 subjects used for our discovery GWAS.

## Results

### Multiple genes are associated with HV and entorhinal thickness in Koreans

**Fig. 1:**
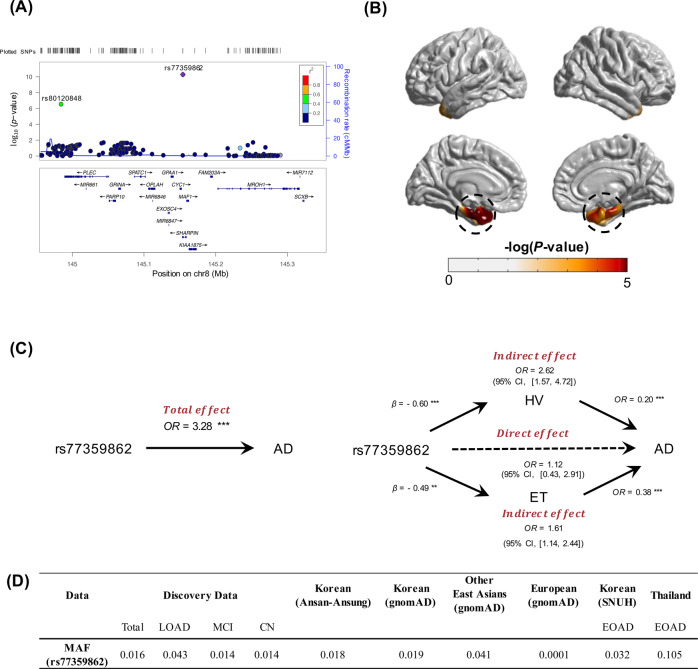
Association of *SHARPIN* missense variant rs77359862 with AD and related traits. **A** Regional plot for the association of *SHARPIN* with hippocampal volume (HV). **B** Illustration of cortical atrophy in left and right hemispheres. A general linear model was applied to detect point-wise differences in whole-brains of 84 carriers and 2559 non-carriers of the rs77359862 missense variant. Rs77359862 carriers (bottom) show greater atrophy in the entorhinal cortex and hippocampus (highlighted in dotted black circles) compared to non-carriers. **C** Mediation analysis shows that the effect of rs77359862 on HV and entorhinal cortical thickness (ET) mediates the association of rs77359862 and AD risk. The total effect represents the impact of rs77359862 on the odds of AD without considering indirect effects of the variant through the hippocampus and entorhinal region, whereas the direct effect is calculated controlling for its effect on HV and ET which are both associated with AD. Each indirect effect was obtained by adjusting for the other mediator; β regression coefficient. OR odds ratio, 95% CI 95% confidence interval of 10,000 bootstraps; **p-value < 0.01, ***p-value < 0.001. **D** Frequency of the rs77359862 missense variant in late-onset AD cases, early-onset AD (EOAD) cases, mild cognitive impairment (MCI) cases, and cognitively normal (CN) persons in clinical samples from Korea and Thailand and in several reference populations. Other East Asians exclude Korean and Japanese. Detailed information for EOAD data is provided in supplementary Text 6. SNUH Seoul National University Hospital.

GWAS conducted for the five sMRI traits revealed GWS (*p* < 5.0 × 10^−8^) and suggestive (*p* < 1.0 × 10^−6^) associations for HV, entorhinal cortical thickness (ET), superior frontal cortical thickness, middle temporal cortical thickness and inferior parietal cortical thickness with SNPs in multiple regions (Supplemental Figs. S3 and S4). We found that the *APOE* genotype was significantly associated with the measures for the entorhinal (*p* = 5.1 × 10^−11^), superior frontal (*p* = 3.9 × 10^−8^), middle temporal (*p* = 5.8 × 10^−7^) and inferior parietal (*p* = 4.2 × 10^−8^) cortex regions, and the HV (*p* = 6.3 × 10^−20^) (Supplemental Table S3). These results are explained almost entirely by the dose-dependent effects of the ε4 allele on AD risk compared to the ε3ε3 reference genotype. The ε2 allele was protective but this effect was not significant, potentially due to the low frequency of this allele in Koreans. GWS associations were also observed for a missense variant (rs77359862) in *SHARPIN* with decreased ET (*β* = 0.59, *p* = 5.0 × 10^−9^) and HV (*β* = 0.62, *p* = 5.1 × 10^−12^) even after accounting for the *APOE* genotype (Table 1). Rs80120848, located ~5 kb apart from *PLEC*, was also associated with HV at the GWS level (*p* = 2.3 × 10^−8^, *β* = 0.53). Rs80120848 is 189 kb apart from and moderately correlated with rs77359862 (*r* = 0.68). To determine whether these are independent association signals, we tested a conditional model including both variants, and found that rs80120848 was not associated with HV (*p* = 0.32), whereas the association with rs77359862 was still significant (*p* = 4.2 × 10^−^^4^) indicating that these effects may not be independent association signals and are most likely driven by the *SHARPIN* locus (Fig. 1A). Suggestive associations were observed for ET with two SNPs (rs7160806, *p* = 7.1 × 10^−7^; rs1956822, *p* = 5.8 × 10^−7^) located in *NOVA-AS1* that encodes a long intergenic non-protein-coding RNA 2588 and for HV with rs150912768 (*p* = 6.9 × 10^−7^) located in *LOC1053722*, a gene of unknown function that has an overlapping but reversely transcribed start site with *SMAD4*. Genome-wide analyses that were not adjusted for the *APOE* genotype did not reveal any additional GWS or suggestive associations outside of the *APOE* region (Supplementary Table S4). Our GWAS results for previously identified SNPs from other cohorts are provided as supplementary information (Supplementary Text 4, Supplementary Table S5).

**Table 1:** Significant GWAS results (*p* < 1.0 × 10^−6^) for entorhinal cortical thickness and hippocampal volume adjusted for *APOE* genotype.

Trait	Chr	Position	SNP	MA	MAF	IQS	*β*	SE	*p*-value	Locus
Entorhinal thickness	8	145154282	rs77359862	A	0.01	G	−0.59	0.10	5.0 × 10^−9^	*SHARPIN*
	14	27221601	rs7160806	G	0.39	0.992	−0.13	0.02	7.1 × 10^−7^	*NOVA1-AS1*
	14	27219914	rs1956822	G	0.39	0.995	−0.13	0.02	5.8 × 10^−7^	*NOVA1-AS1*
Hippocampal volume	8	145154282	rs77359862	A	0.01	G	−0.62	0.09	5.1 × 10^−12^	*SHARPIN*
	8	144984345	rs80120848	A	0.01	G	−0.53	0.10	2.3 × 10^−8^	*EPPK1*/*PLEC*
	18	48554594	rs150912768	T	0.01	0.953	−0.45	0.09	6.9 × 10^−^7	*SMAD4/ELAC1*

*Chr* chromosome, *MA* minor allele, *MAF* minor allele frequency, *IQS* imputation quality score, *G* genotyped SNP, *SE* standard error

Gene-based analyses of 18,950 protein-coding genes including rare variants with minor allele frequencies (MAF) < 0.01 after adjusting for the *APOE* genotype revealed several gene-wide significant (2.6 × 10^−6^) associations with HV and ET (Supplementary Fig. S5a) and little evidence for genomic inflation (Supplementary Fig. S5b) including *COX7A2L* (234 SNPs, *p* = 1.9×10^−6^) with ET and genes with HV: *GUCA1A* (64 SNPs, *p* = 7.7 × 10^−7^), *VIT* (289 SNPs, *p* = 7.8 × 10^−9^), and *METTL6* (163 SNPs, *p* = 2.0 × 10^−6^). The association of HV with *GABRR2* almost reached the gene-wide significance threshold (108 SNPs, *p* = 3.2 × 10^−6^).

### *SHARPIN* missense variant rs77359862 indirectly affects AD risk through its impact on AD-specific brain damages

To evaluate the effect of rs77359862 on cortical atrophy, cortical thickness measures derived from multiple points spanning the entire cortex were separately regressed on rs77359862 with a generalized linear model (GLM). The 84 carriers of the rs77359862 missense variant including 28 CN subjects, 40 MCI, and 16 AD patients displayed significantly greater atrophy in the entorhinal cortex and hippocampus, than the 2559 non-carriers, whereas the differences between carriers and non-carriers were not significantly different in any other cortical regions (Fig. 1B) (see Supplementary Text 5 for Methods). Next, we conducted mediation analysis to estimate the indirect effect of rs77359862 on AD risk through its impact on HV and ET. As shown in Fig. 1C, rs77359862 is significantly associated with AD (total effect, OR = 3.28*, p* = 1.2 × 10^−4^), but after controlling for its association with HV and ET, the strength of this relationship was attenuated (OR = 1.12*, p* = 0.82), suggesting that the mechanism underlying the effect of rs77359862 on AD risk is mediated by its direct contribution to neurodegeneration particularly in the hippocampal and entorhinal cortex regions. Routes through the hippocampus and entorhinal cortex account for 67% and 33%, respectively of the indirect effect of rs77359862 on AD risk. Consistent with the finding of an attenuated effect of rs77359862 on AD risk after adjusting for the indirect routes, the rs77359862 minor allele increases AD risk 2.62-fold (CI: [1.57, 4.72]) via the hippocampus and 1.61-fold (CI: [1.14, 2.44]) via the entorhinal cortex.

### *SHARPIN* missense variant rs77359862 is associated with AD-related clinical measures and biomarkers

**Fig. 2:**
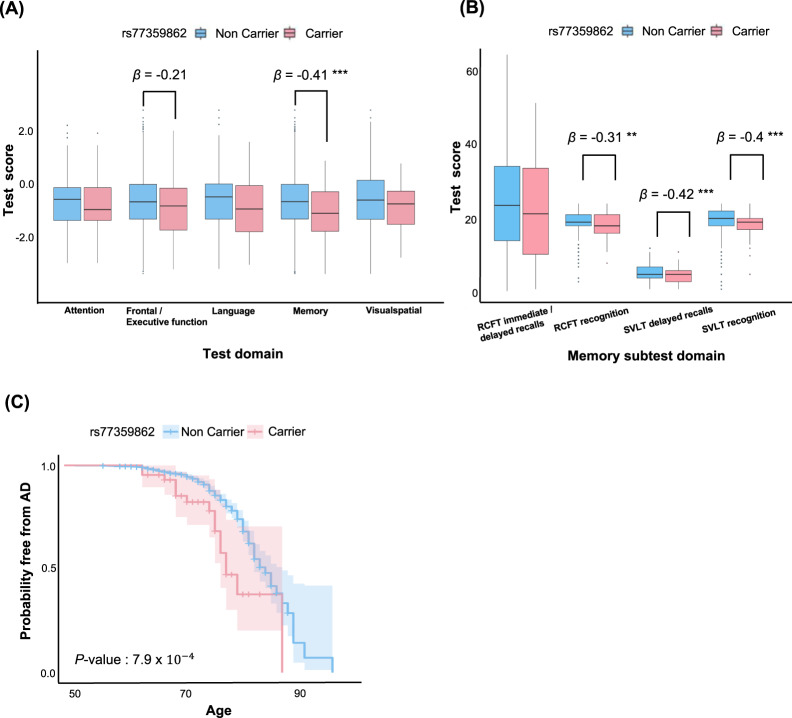
Association of rs77359862 with brain imaging, cognitive test measures and AD onset. **A** Boxplots of Neuropsychological test (Seoul Neuropsychological Screening Battery (SNSB)) scores for attention, executive function, language, memory and visuospatial ability among rs77359862 carriers and non-carriers. Rs77359862 carriers had significantly lower scores for executive function and memory. **p-value < 0.01, ***p-value < 0.001. **B** Boxplots of subtests of memory domain in SNSB. Four subtests of memory function were assessed including visual memory (Rey-Complex Figure Test (RCFT) immediate/delayed recalls and recognition) and verbal memory (Seoul Verbal Learning Test (SVLT) delayed recalls and recognition). Rs77359862 carriers had significantly lower scores for all subtests, except RCFT immediate/delayed recalls. **p-value < 0.01, ***p-value < 0.001. **C** The rs77359862 missense variant significantly lowers age of AD onset. The effect of rs77359862 genotype on onset age was evaluated using Kaplan–Meier analysis and the survival curves for carriers and non-carriers of the missense variants were compared using the log-rank test.

We evaluated the association of the rs77359862 missense variant and multiple measures of cognitive function using linear regression models that included covariates for age and sex. Significant associations were observed in measures of memory (*β* = −0.41, *p* = 1.0 × 10^−4^) and, to a lesser degree, in frontal/executive function (*β* = −0.21*, p* = 0.04), but not in attention (*β* = −0.09*, p* = 0.38), language (*β* = −0.18*, p* = 0.09) or visuospatial ability (*β* = −0.10*, p* = 0.33, Fig. 2A). Four subtests in memory measurement include Rey-complex Figure Test (RCFT) immediate/delayed recalls (*β* = −0.31*, p* = 0.16), RCFT recognition (*β* = −0.31*, p* = 0.006), Seoul Verbal Learning Test (SVLT) delayed recalls (*β* = −0.42*, p* = 1.6 × 10^−4^), and SVLT recognition (*β* = −0.40*, p* = 4.1 × 10^−4^) (Fig. 2B). Next, we investigated the effect of rs77359862 on age of AD symptom onset using the Kaplan–Meier approach to estimate a survival curve. This analysis showed that AD onset among individuals with the rs77359862 mutant variant was on average 1.5 years earlier than among those with the *G* allele (log-rank test *p* = 7.9 × 10^−4^, Fig. 2C).

In a subset of 876 subjects who were classified as CN or MCI at baseline and followed longitudinally (on average for 28.8 months), we also examined the impact of rs77359862 on the progression across clinical stages leading to AD. Within this group, 53 CN (10.1% of the CN) and 21 MCI participants (6.9% of the MCI) converted to MCI and AD, respectively (8.4% of the total) (Supplemental Table S6). The frequency of the mutant allele among the converters (6/74 = 8.1%) was higher than among the non-converters (26/802 = 3.2%). Analysis of the effect of rs77359862 genotype on the likelihood of conversion using a proportional hazards model adjusting for *APOE* genotype showed that participants with the mutant allele were 2.66 times as likely to progress to the next stage of cognitive decline (*p* = 0.023).

Analysis of the association of rs77359862 and Aβ accumulation in the brain using a logistic regression model with covariates for age and sex demonstrated that carriers of the rs77359862 missense variant had greater Aβ accumulation than non-carriers (*p* = 0.02, OR = 1.84).

### Association of *SHARPIN* missense variant rs77359862 with HV in other cohorts

In our study, the frequency of the rs77359862 missense variant was consistently over 1% in CN Koreans in our study (1.4%) and in the Ansan-Ansung cohort (1.8%) [37], as well as in other East Asians (other than Korean and Japanese), included in the gnomAD database (4.1%) [38] (Fig. 1D). We observed a higher frequency of the variant among Koreans with late-onset AD (LOAD) in our study (4.3%) and in 77 early-onset AD (EOAD) patients who were diagnosed at the Seoul National University Bundang Hospital (3.2%). This variant was also more prevalent in a sample of Thailand EOAD patients (10.5%) [39]. Detailed information of EOAD patients was provided in supplementary Text 6. In contrast, the rs77359862 missense variant was virtually absent in non-Finnish persons of European ancestry (MAF = 0.0001) [38]. Due to rarity, we were unable to evaluate the rs77359862/AD association in populations with European ancestry. Therefore, we hypothesized that other variants in *SHARPIN* may be associated with MRI traits and AD in non-Asians. We conducted gene-based analyses by testing the association of HV with *SHARPIN* including 20 kb beyond the gene boundaries using imputed GWAS data from the ADNI [29] and AddNeuroMed cohorts [30]. Gene-based analyses revealed significant associations with *SHARPIN* in both the ADNI (86 SNPs, *p* = 0.002) and AddNeuroMed (93 SNPs, *p* = 0.04) datasets. Other suggested significant variants with HV from Table 1 were not significant in the ADNI dataset (EPPK1: 35 SNPs, *p* = 0.05; PLEC: 169 SNPs, *p* = 0.58; SMAD4: 130 SNPs, *p* = 0.52; ELAC1: 24 SNPs, *p* = 0.24).

### Rs77359862 missense variant alters the stability of SHARPIN complex structure

**Fig. 3:**
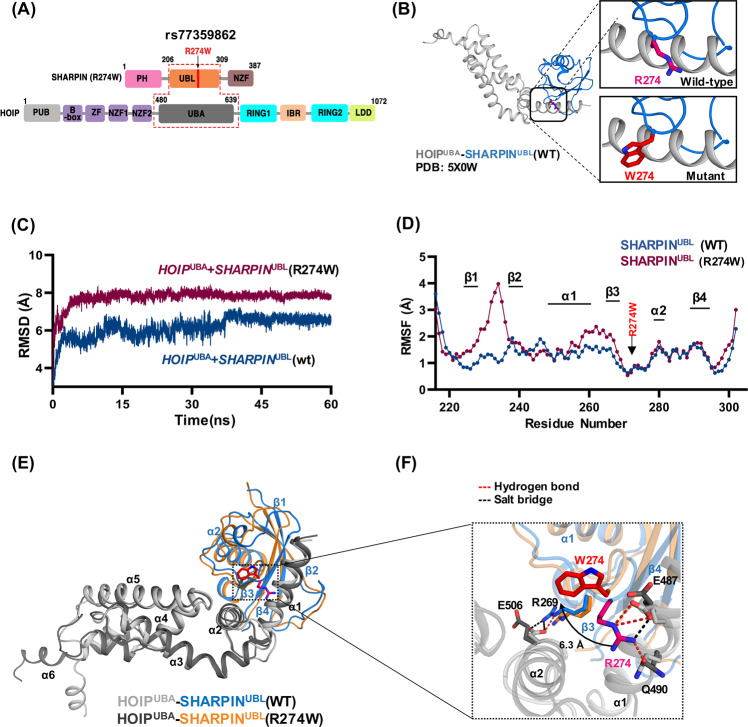
Molecular dynamics simulation modeling of the effect of the SHARPIN R274W mutation on the HOIP^UBA^-SHARPIN^UBL^ complex. **A** Domain map of SHARPIN and HOIP proteins. Protein binding occurs at the HOIP^UBA^ and SHARPIN^UBL^ domains. **B** Crystal structure of the HOIP^UBA^-SHARPIN^UBL^ (PDB: 5X0W) binding complex indicating the location of the SHARPIN R274 residue in the wild type (WT) and manually mutated W274. **C** The root mean square deviation (RMSD) plot indicates the overall global deviation of the protein complex during 60 ns in WT and mutant. **D** The root mean square fluctuation (RMSF) plot shows the fluctuations of each residue of the SHARPIN (R274W) in complex during the last 20 ns of the equilibrated run. The structural elements corresponding to those residues were indicated above the plot. **E** Structural alignment of the structures obtained by averaging the atomic coordinates of the last 20 ns of the simulated run between WT complex and the mutant complex. The color code is maintained throughout the figure as per the legend between HOIP^UBA^-SHARPIN^UBL^ WT and R274W mutant protein complexes which are represented as cartoons and the residues in stick representation. The structural elements were marked starting from N-terminal ends of the protein. **F** The mutation site region is zoomed in from (**E**) (indicated by rectangular box) and the break in salt bridge (black dashed line) and hydrogen bonds (red dashed line) just with respect to the mutation is shown. The deviation of the residue from the WT to the mutant is marked by the arrow and the distance mentioned in Å.

The rs77359862 variant is located in the domain mediating the binding of SHARPIN to its ligand HOIL-1-interacting protein (HOIP), which encodes the RING-between-RING (RBR) domain type ε3 ligase. The binding sites of these two proteins are the HOIP N-terminal UBA domain (HOIP^UBA^) and the UBL domain of SHARPIN (SHARPIN^UBL^) [40] (Fig. 3A). The variant of rs77359862 located in SHARPIN^UBL^ leads to the substitution of polar R274 to hydrophobic tryptophan (NP_112236.3:p.R274W) (Fig. 3B). This switch in the chemical properties of the amino acids at the interface seems to affect the stability of the bound HOIP^UBA^-SHARPIN^UBL^ complex. To understand the effect of this variant, we performed a molecular dynamic (MD) simulation for the wild-type (WT) complex (PDB:5X0W) and an in silico SHARPIN mutated (R274W) complex. As shown in Fig. 3C, over time, the global root mean square deviation (RMSD) value of the mutant HOIP^UBA^-SHARPIN^UBL^(R274W) was ~2 Å higher than the WT complex over time mainly due to the fluctuation in structural elements, such as the loops between β1-β2 and α1-β3 of the mutant SHARPIN^UBL^ (R274W) complex (Fig. 3D). Accordingly, amino acids in those regions were highly variable compared to those in the other parts of the protein as deduced by the root mean square fluctuation (RMSF) plot.

Interaction on the interface between HOIP^UBA^ WT and SHARPIN^UBL^ WT is strengthened by residues that contribute to hydrogen bonds and salt bridges [40]. Upon 60 ns MD simulation, we compared structural changes by using the averaged atomic coordinates for the stabilized last 20 ns (Fig. 3E, F). The comparative analysis revealed that the WT complex interface was built with ten hydrogen bonds and seven salt bridges, while the number of hydrogen bonds and salt bridges in the mutant complex HOIP^UBA^-SHARPIN^UBL^ (R274W) had decreased to 4 and 0, respectively (Supplemental Table S7), indicating that the mutant in SHARPIN induced a weaker interaction between the HOIP^UBA^ and SHARPIN^UBL^ (R274W) (Fig. 3F and supplemental Fig. S6) (detailed information is provided in supplementary Text 7 and supplemental Figs. S7–8).

**Fig. 4:**
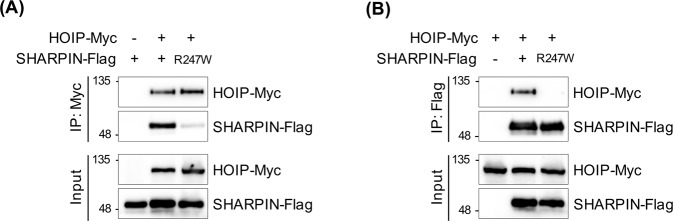
Effect of mutant SHARPIN (R274W) on binding with HOIP. 293T cells transiently co-transfected with Flag-tagged WT and mutant SHARPIN and Myc-tagged HOIP were immunoprecipitated using anti-Myc antibody (**A**) and anti-Flag antibody (**B**). The binding affinity between SHARPIN variants and HOIP was confirmed through western blot analysis using anti-Myc and anti-Flag antibody, respectively.

These observations were further supported by co-immunoprecipitation (co-IP) experiments (the detailed method is provided in supplementary Text 8). To determine whether the SHARPIN mutant R274W affected interactions with HOIP, flag-tagged SHARPIN WT and R274W mutant were co-immunoprecipitated with Myc-tagged HOIP WT (Fig. 4A) and Myc-tagged HOIP WT was co-immunoprecipitated with flag-tagged SHARPIN WT and R274W (Fig. 4B). The binding between SHARPIN^UBL^ (R274W) and HOIP^UBA^ was significantly reduced compared with that of SHARPIN WT.

In summary, our studies revealed that the SHARPIN mutant R274W might render the interaction between HOIP^UBA^ and SHARPIN^UBL^ unstable, and thus destabilize the downstream SHARPIN-mediated pathway.

## Discussion

Previous GWAS have identified many GWS loci for AD risk with GWS, but they have not been consistently replicated [7, 8, 15, 18, 41, 42]. The case/control study design has multiple limitations, including the power to detect associations with variants conferring small effects and biological complexity underlying, even well-defined, disease phenotypes. Genetic studies of AD are further complicated by mis-diagnosis, phenotypic heterogeneity (e.g., co-existing cerebrovascular disease and other brain pathologies, variable deficits in memory, language, and executive function), and misclassification of “controls” who may develop AD later in life. Studies of AD-related endophenotypes offer several advantages to gene discovery because statistical power is greater for quantitative traits compared to dichotomous outcomes, are not subject to misclassification, and are likely to have a less complex genetic architecture. Although our study is much smaller than previous GWAS of brain MRI traits conducted in population-based cohorts [43], our sample includes relatively larger AD and MCI cases, compared to CN cases, and significant associations with structural brain changes are more likely related to AD than normal aging.

Kang et al. performed GWASs for cases/controls with AD (*n* = 2291) in a Korean sample [44], but novel AD-related loci were not discovered. However, we identified a GWS association of a missense variant (rs77359862) in *SHARPIN* with decreasing ET and HV in a Korean population. This variant is infrequent in Koreans (MAF = 0.018), and virtually absent in populations outside of East Asia. Hence, we were unable to replicate this finding using non-Asian datasets. However, we found significant associations of HV with other rare and infrequent functional *SHARPIN* variants evaluated using a gene-based test in two large European ancestries (ADNI and AddNeuroMed) cohorts. Furthermore, Soheili-Nezhad et al. found a GWS association between another *SHARPIN* non-synonymous variant (rs34173062), located 4,325 base pairs away from rs77359862 and very rare in Koreans (MAF = 0.00035), and a composite MRI measure of limbic degeneration in the ADNI cohort (*p* = 2.1 × 10^−10^) [45]. The same variant was significantly associated with the thickness of the left entorhinal cortex (*p* = 0.002), right entorhinal cortex (8.6 × 10^−4^), and a history of AD in both parents (*p* = 2.3 × 10^−6^) in a UK Biobank (UKB) cohort dataset (*n* = 8428) [45]. Another recent large GWAS (*n* = 409,435) that included UKB data identified a GWS association between AD and yet another *SHARPIN* missense variant (rs34674752, *p* = 1.0 × 10^−9^) [46]. These results suggest that *SHARPIN* contributes to the development of AD in Koreans and Caucasians of European ancestry.

Mediation analysis findings suggest that the *SHARPIN* rs77359862 variant increases the risk of AD more than threefold, primarily through its effects on the entorhinal cortex and hippocampus. Our PET findings indicate that the same variant also affects the accumulation of Aβ, the main component of the amyloid plaques observed in PET images, which is a cardinal pathological feature of AD and is functionally related to frontal and memory regions [47]. According to Jung et al. [48], decreased executive function and memory, but not language or visuospatial impairment, were associated with a higher risk of cognitive decline, which is consistent with our observation that rs77359862 was significantly associated with executive and memory, but not language, visuospatial or attentional abilities. Longitudinal follow-up in this study revealed that CN and MCI carriers of the rs77359862 missense variant were significantly more likely to progress to MCI and AD, respectively. Finally, previous studies have shown that variants in several genes (e.g., *ABCA7, SORL1, TREM2*) are associated with both early-onset and late-onset AD [39]. The EOAD-associated variants are rare (MAF«0.01) and the LOAD-associated variants are infrequent (MAF > 0.01) or common (MAF > 0.05) in the populations in which they occur. With the notable example of carriers of a rare and highly penetrant mutation of the presenilin 2 gene, who developed AD symptoms between 45 and 88 years old [49], where we observed one of a few documented instances of a variant (rs77359862) that is associated with both EOAD and LOAD.

SHARPIN is a component of the linear ubiquitination assembly complex (LUBAC) [50] and, together with HOIP, suppresses NF-κB signaling [51, 52]. To study the mutational effect (R274W) of SHARPIN^UBL^ on its complex formation with HOIP^UBA^, we performed a 60 ns MD simulation using the crystal structure [40] (PDB: 5X0W), for both WT and mutant variants (R274W). Our MD analysis strongly suggests that the mutant complex HOIP^UBA^-SHARPIN^UBL^(R274W) destabilizes the complex at the interface, as preserving the minimal integrity of the complex structure, which was validated with co-IP experiments. Therefore, the physical interaction between HOIP and R274W mutant SHARPIN was significantly reduced compared to the interaction with SHARPIN WT, and this unstable complex did not affect the downstream NF-κB signaling pathway [53]. NF-κB function in primary microglia isolated from *SHARPIN* mutant mice, rather than using an overexpression system will be evaluated in our future work.

NF-κB signaling in the nervous system plays a crucial role in the pathophysiology of AD, including neuroinflammation, deficits in memory consolidation, Aβ clearance, and neuronal cell death [54]. A rare functional variant (rs572750141, NM_030974.3:p.Gly186Arg) of *SHARPIN* has previously been found in a Japanese population and is significantly associated with an increased risk of LOAD [16]. This mutant of *SHARPIN* showed reduced NF-κB activation in HEK293 cells. SHARPIN is enriched at synaptic sites in mature neurons where it colocalizes with SHANK1 [55]. It is well known that activated NF-κB can be transported from activated synapses to the soma, which is essential for long-term memory. The reduction of neuronal NF-κB activity by the *SHARPIN* variant can inhibit anti-apoptosis pathways and lead to apoptosis or necroptosis in neurons [54, 56]. A recent study reported that knocking down SHARPIN using siRNA, inhibits Aβ-induced phagocytosis in macrophages [57], supporting our result of a marked increase in the accumulation of amyloid plaques in subjects with the R274W mutant SHARPIN.

There is mounting evidence that rare variants (MAF < 0.01) have large effects on AD risk [58–60] and may account for much of the missing heritability of the disorder [5]. We identified significant associations with several novel genes (*COX7A2L*, *GUCA1A, VIT*, *GABRR2*, and *METTL6*) through gene-based tests of aggregated rare variants with predicted functional consequences. Mitochondrial dysfunction has been widely reported in AD [61–63]. It has been reported that AD patients have a deficit of cytochrome C oxidase (*COX*) in both peripheral and brain tissue [64]. mRNA levels of several Cox genes including Cox7a2 correlated significantly with the Aβ plaque burden in the hippocampus of an AD mouse model [65]. *VIT* is known to be involved in brain asymmetry [66]. Gamma-aminobutyric acid (*GABA)*, the major inhibitory neurotransmitter in the brain, is widely distributed in neurons of the cortex and contributes to many cortical functions by binding to GABA receptors, ligand-gated chloride channels. There is a large body of evidence suggesting that expression of the GABA receptor subunit alpha 1 gene (*GABRA1*) receptor gene is altered in the hippocampus of AD cases [67]. A SNP in the gene encoding GABA receptor subunit rho-2 (*GABRR2*) was associated with the general cognitive ability [68]. *METTL6* was identified as a marker of the proliferation of luminal breast cancers [69, 70], but the biological role of this RNA-modifying methyltransferase is not well understood.

Our study has several limitations. The sample had limited power to detect genome-wide significant associations for rare variants with small effects, and this concern was exacerbated by the smaller number of AD cases compared to MCI cases and control subjects. However, the sample was adequately powered to detect larger effects, evidenced by the GWS association with the *SHARPIN* missense variant. In addition, longitudinal brain MRI data were available for only a small portion of individuals, thus limiting our ability to perform genome-wide scans for atrophy in brain measures, over time. Furthermore, experiments in cell or mouse models will need to be conducted to demonstrate the effects of rs77359862 on AD-specific pathological damages such as Aβ accumulation or tangle formation.

## Supplementary information

Supplementary Text

Supplementary Figure legends and Table tiles

Supplemental Figure1

Supplemental Figure2

Supplemental Figure3

Supplemental Figure4

Supplemental Figure5

Supplemental Figure6

Supplemental Figure7

Supplemental Figure8
