# Supplementary material for: A missense variant in SHARPIN mediates Alzheimer’s disease-specific brain damages
Source: Transl Psychiatry. 2021 Nov 16;11:590. doi: 10.1038/s41398-021-01680-5 (PMC8595886; doi:10.1038/s41398-021-01680-5)
Supplement: Supplementary file 1 — Supplementary Text [file 41398_2021_1680_MOESM1_ESM.docx]

**A missense variant in *SHARPIN* mediates**

**Alzheimer’s disease-specific brain damages**

**Text S1. Genome-wide Association Study Participants**

The 5,570 subjects of study sample underwent neuropsychological assessment yielding a Clinical Dementia Rating (CDR) score and a sMRI scan. The clinical diagnosis of AD status designated according to criteria issued by the National Institute of Neurological and Communicative Disorders and Stroke–Alzheimer Disease and Research Disorders Association (NINCDS-ADRDA) criteria [1]. Cognitively normal (CN) subjects had no evidence of neurological disease or impairment in cognitive function or activities of daily living. Subjects with focal lesion detected by the brain MRI scan, history of head trauma, or psychiatric disorder that could affect mental function were excluded.

**Text S2. Genotyping, quality control, imputation, and principal component analysis procedures**

A total of 5,570 subjects were genotyped using an Affymetrix customized KoreanChip[2, 3]. Genotype data were processed using PLINK[4] and ONETOOL[5]. SNPs were eliminated if the genotype call rate was < 95%, not in Hardy-Weinberg equilibrium (*p* < 1×10^-5^ )[6], or if there was a significant difference (*p* < 1×10^-5^) in call rate between the CN, MCI and AD subjects. Subjects were excluded if they were duplicated (identity-by-state > 0.9), the X-chromosome homozygosity was between 0.2 and 0.8 or not consistent with phenotypic sex, *APOE* genotype was missing, the genotype call rate was < 95%, SNP heterozygosity rate greater than three standard deviations from the average heterozygosity rate, or considered an outlier by analysis of principal components (PC) of ancestry. After applying these filters, 4,562 subjects and 685,742 SNPs remained.

Genotypes were pre-phased using SHAPEIT [7] and then imputed using the 1000 Genomes Phase 3 reference panel and IMPUTE2 [8]. Imputed SNPs were excluded if the INFO score < 0.5, genotype call rate <0.98, or *p-*value for HWE <1×10^-6^. As a result, 4,562 subjects and 13,715,061 SNPs remained. The detailed procedure for quality control is illustrated in Supplemental Figure S1. Analysis of principal components of ancestry (PCs) showed that there is little evidence of population stratification (Supplemental Figure S2).

**Text S3. Brain MRI acquisition and processing**

T1-weighted images (Siemens Healtheneers, Erlangen, Germany) were obtained as previously described [9] and preprocessed with FreeSurfer [10] V.5.3. The AD-related traits selected for GWAS included measures of hippocampal volume and thickness of the entorhinal, inferior parietal, middle temporal, and superior frontal regions. of the cortex MRI traits were available for 209 AD, 1,449 MCI, and 985 CN subjects with genotype data. We observed non-normal trait distributions and substantial trait distribution differences between subjects measured with 3.0 tesla (T) (n=1,955) scanners (Skyra, Siemens, TR = 2300 ms; TE = 2.143 ms; TI = 900 ms; 9 flip angle; FoV = 256x256; matrix = 320x320; number of slices = 178) compared to 1.5 T (n=688) scanners (Magnetom Avanto, Siemens, TR = 1800 ms; TE = 3.43 ms; TI = 1100 ms; 15 flip angle; FoV = 224x224; matrix = 256x256; number of slices = 176). Hence, traits for each subgroup were standardized and then transformed by an inverse normal transformation. Descriptive statistics for the MRI traits (Supplementary Table S1) were obtained using Rex Version 3.0.3 software (RexSoft Inc., Seoul, Korea).

**Text S4. Comparison of Genome-wide Association Study results with previous researches**

Twenty-five susceptibility loci, which had been identified by previous GWASs, were analyzed in out GWAS results (Supplementary Table S5) [11-16]. When we could not obtain SNPs from our dataset, the closet, and the highest level of linkage disequilibrium loci from the 1000 Genomes Project were selected as a replacement. It was noted that previous GWASs for AD were performed for cases/controls with AD in the Western population, but ours were for the hippocampus volume in Korean. Rs3752246 on ABCA7 showed significant association with HV atrophy (*p*=3.9x10^-5^, β=-0.1), but others did not.

**Text S5. Whole-brain neuroimaging analysis with rs77359862**

The effect of the GWS *SHARPIN* SNP associated with MRI traits was further assessed for their influences on whole-brain cortical thickness measures. The SNP genotype effect was analyzed using a dominant model. We applied GLM to infer point-wise cortical atrophy with the SurfStat toolbox (<http://www.math.mcgill.ca/keith/surfstat/>) implemented in MATLAB (R2012a, The Mathworks, Natick, MA, USA), while using for age, sex, *APOE* ε4 status, and field strength as covariates. A random filed theory (RFT)-based correction was applied to correct for multiple point-wise cortical thickness comparisons [17].

**Text S6. Early-onset AD patients**

For MAF of rs77359862, data for 77 subjects with early-onset AD (EOAD) were provided by Seoul National University Bundang Hospital (SNUBH). The average age of onset among these subjects was 57 years. An EOAD patient sample of five males and 14 females with an average of onset of 55 years was recruited at the Department for Neurodegenerative Diseases, Center for Neurology, Thailand [18]. The study of the Thai EOAD sample was approved from the Faculty of Medicine Siriraj Hospital, Mahidol University, Bangkok, Thailand.

**Text S7. Molecular dynamics (MD) simulation and analysis**

Initially, R274 of SHARPIN (^SHARPIN^R274) forms a salt bridge with ^HOIP^E487 and a hydrogen bond with ^HOIP^Q490 in the WT complex, while the mutation R274W breaks the salt bridges (^SHARPIN^R274-^HOIP^E487) and the hydrogen bonds (^SHARPIN^R274-^HOIP^Q490 and ^SHARPIN^R274-^HOIP^E487) (Figure 3d). The residues (^HOIP^E487 and ^HOIP^Q490), interacting with ^SHARPIN^R274 in the WT complex, reside on the α1 helix of HOIP^UBA^. Therefore, the removal of the interactions is likely to induce a series of conformational changes in other parts of the protein, which in turn could destabilize the mutant complex. The immediate structural effect of this mutation would be the breakage of a salt bridge between ^HOIP^E506 on α2 and ^SHARPIN^R269 on β3, that is located proximal to the mutation site.

In the line of structural destabilization events of Figure 3c and d, remarkably, the helix α1 of HOIP^UBA^ was disassembled by three amino acids (^HOIP^R496-G498) at the C-terminus, which subsequently transformed into a loop in the HOIP^UBA^-SHARPIN^UBL^ (R274W) complex (Supplemental Figure S6c). The consequence of the disassembly led to the breakage of a hydrogen bond and a salt bridge between α1 of HOIP^UBA^ and a loop (D227-A238) of SHARPIN^UBL^ (R274W), which weakened the intermolecular interactions in the mutant complex (Supplemental Figure S6c left). Additionally, the hydrogen bond between ^HOIP^E499 and ^SHARPIN^S301 was broken due to the massive displacement of ^HOIP^E499 by 10.6 Å (Supplemental Figure S6c right). Subsequently, the displacement of the ^HOIP^R496 side chain by ~5 Å leads to a break in the salt bridge with ^SHARPIN^E226 in the mutant complex and a hydrogen bond with ^SHARPIN^S230 (Supplemental Figure S6c right).

Intriguingly, the loss of the hydrogen bond and salt bridge by the mutation in SHARPIN^UBL^ (R274W) possibly tilted the N-terminal half of the α1 helix toward SHARPIN^UBL^ (R274W) by ~ 2.2 Å at ^HOIP^Q481 (Supplemental Figure S6a). This movement induced additional loss of hydrogen bonds between α1 and β4, where the interaction between ^HOIP^R485 on α1 and ^SHARPIN^P294 on β4 was removed. However, the hydrogen bond between ^HOIP^Q481 at the N-terminal end of the α1 and ^SHARPIN^D293 on β4 was preserved because the two secondary elements had moved in parallel **(**Supplemental Figure S6a right), which might help the minimal integrity of the complex structure. Despite the decreased number of interactions in the mutant complex, the complex's interface seemed to maintain the integrity of the complex structure containing SHARPIN^UBL^ (R274W) by hydrophobic interactions (Supplemental Figure S6b). Indeed, this hydrophobic patch was found in the α1 of HOIP^UBA^ and β4 and a loop (V271-L283) in SHARPIN^UBL^ (R274W) (Supplemental Figure S6b right). Therefore, these reduced number of hydrogen bonds, salt bridges, and hydrophobic interactions, possibly led to the conformational changes in the mutant R274W that destabilized the interface between HOIP^UBA^ and SHARPIN^UBL^ (R274W) complex.

The side chain characteristics of the R274W mutation, in which the positively charged arginine is replaced with a non-polar tryptophan could reverse the electrostatic potential at the binding interface (Supplemental Figure S7). The resulting electrical charge on the interface became similar for both proteins, conceivably weakening the electrostatic interaction required for protein-protein complex formation. Therefore, these observations imply that the reduction of hydrogen bonds and salt bridges together with the reversed electrostatic property probably destabilize and separate the HOIP^UBA^-SHARPIN^UBL^(R274W) complex during the simulation. In contrast, the complex persists even after 60 ns, although the interactions appeared weakened. In Supplemental Figure S8, a change in the hydrophobic patch between the two proteins was observed in the simulated mutant model.

MD simulation was performed using the crystal structure of SHARPIN^UBL^ bound to its ligand HOIP N-terminal UBA domain (HOIP^UBA^) (PDB ID: 5X0W [19]). The missing residues in SHARPIN^UBL^ (Ala235) and HOIP^UBA^ (Gly589-Gly593) in the crystal structure were modeled using the reference SHARPIN sequences and Modweb version r214 in Chimera1.13.1 [20]. Selenomethionine residues from the crystal structure were replaced with methionine using CHARMM-GUI [21]. The mutant variant, R274W, was obtained by manually mutating the Arg274 residue of SHARPIN^UBL^ (from PDB: 5X0W) to Trp (R274W) using the Pymol v2.3 [22] mutagenesis function. To set up the simulation system, SHARPIN^UBL^ (WT) and SHARPIN^UBL^(R274W) complexed with HOIP^UBA^ was solvated by TIP3P water in a PBC rectangular box with a minimum of 10 Å box-padding and neutralized by 0.15 M NaCl. Following the annealing for 12,000 steps, both the WT and mutant complexes were set for energy minimization at 0 K temperature for 10,000 steps to reach a temperature of 310 K. Subsequently, a 200-ps equilibration step was run to distribute heat and restrain the carbon backbone. Finally, the MD production simulation was run for 60 ns in NPT ensemble using the CHARMM27 [23] force field in the NAMD2.13 package[24]. The MD trajectories obtained from each simulation were saved in 40 ps intervals and analyzed using VMD [25], an in-house TCL script, and Bio3D [26] packages in RGui-3.6.1. The last stable 20 ns of both WT and mutant were averaged and compared for analysis. The electrostatic potential of the model was calculated using the APBS suite in Pymol v2.3[22].

**Text S8. Immunoprecipitation (IP) and Immunoblotting**

pCMV3flag8SHARPIN (#50014) and HOIP ORF clone (#RC204117) plasmids were purchased from addgene (#50014, Addgene, MA, USA) and origene (#RC204117, MD, USA), respectively. HOIP was cloned into pcDNA6/myc-His A. The mutant SHARPIN R247W was constructed by site-directed mutagenesis. SHARPIN WT and SHARPIN R247W vectors were transfected with HOIP-myc vector using transfectin (#170-3351; Bio-Rad, CA, USA) into 293T cells. After 36 h, cells were lysed for 1 h at 4°C in IP lysis buffer: 30 mM Tris-Cl (pH 7.4), 150 mM NaCl, 1% Triton-X100, 1 mM Na_3_VO_4_, 50 mM NaF, 1 mM PMSF, 10% glycerol and 2 mM EDTA. For immunoprecipitations, 1 mg of cell extracts were incubated with 1 μg of anti-c-Myc 9E10 primary antibodies (sc-40; Santa Cruz Biotechnology, Tx, USA) or anti-Flag M2 primary antibodies (F3165; Sigma-Aldrich, MO, USA) for 16 h at 4°C, after which they were applied to the protein A/G agarose beads (P9203; GenDEPOT, TX, USA) for 2 h. At the end of incubation, samples were washed three times and subjected to immunoblotting.

Lysates were subjected to sodium dodecyl sulfate polyacrylamide gel electrophoresis and then transferred to polyvinylidene difluoride membranes (IPVH00010; Millipore, Billerica, MA). After blocking any non-specific interactions by using blocking solution (5% skim milk in 0.1% TBST) for 1 hr, membranes were probed with primary antibodies (anti-c-Myc 9E10, 1:1,000 or anti-Flag M2 primary antibodies, 1:10,000) for 14 h at 4°C. Membranes were then incubated with horseradish peroxidase-conjugated anti-mouse IgG (ab131368; Abcam, Cambridge, UK) for 2 h at room temperature. Blots were developed using Clarity Western ECL Substrate (1705061; Bio-Rad), and detected by a Fusion Solo S imaging system (VILBER, Collegien, France).

**REFERENCES**

1. McKhann, G.M., et al., *The diagnosis of dementia due to Alzheimer's disease: recommendations from the National Institute on Aging-Alzheimer's Association workgroups on diagnostic guidelines for Alzheimer's disease.* Alzheimers Dement, 2011. **7**(3): p. 263-9.

2. Moon, S., et al., *The Korea Biobank Array: Design and Identification of Coding Variants Associated with Blood Biochemical Traits.* Sci Rep, 2019. **9**(1): p. 1382.

3. Seo, S., et al., *SNP genotype calling and quality control for multi-batch-based studies.* Genes & Genomics, 2019. **41**(8): p. 927-939.

4. Purcell, S., et al., *PLINK: a tool set for whole-genome association and population-based linkage analyses.* Am J Hum Genet, 2007. **81**(3): p. 559-75.

5. Song, Y.E., et al., *ONETOOL for the analysis of family-based big data.* Bioinformatics, 2018. **34**(16): p. 2851-2853.

6. Wigginton, J.E., D.J. Cutler, and G.R. Abecasis, *A Note on Exact Tests of Hardy-Weinberg Equilibrium.* The American Journal of Human Genetics, 2005. **76**(5): p. 887-893.

7. Delaneau, O., et al., *Integrating sequence and array data to create an improved 1000 Genomes Project haplotype reference panel.* Nature Communications, 2014. **5**(1): p. 3934.

8. Howie, B.N., P. Donnelly, and J. Marchini, *A Flexible and Accurate Genotype Imputation Method for the Next Generation of Genome-Wide Association Studies.* PLOS Genetics, 2009. **5**(6): p. e1000529.

9. Choi, K.Y., et al. *APOE Promoter Polymorphism-219T/G is an Effect Modifier of the Influence of APOE ε4 on Alzheimer's Disease Risk in a Multiracial Sample*. Journal of clinical medicine, 2019. **8**, DOI: 10.3390/jcm8081236.

10. Fischl, B., *FreeSurfer.* Neuroimage, 2012. **62**(2): p. 774-81.

11. Li, J.Q., et al., *GWAS-Linked Loci and Neuroimaging Measures in Alzheimer's Disease.* Mol Neurobiol, 2017. **54**(1): p. 146-153.

12. Shen, L. and J. Jia, *An Overview of Genome-Wide Association Studies in Alzheimer's Disease.* Neurosci Bull, 2016. **32**(2): p. 183-90.

13. Jun, G., et al., *PLXNA4 is associated with Alzheimer disease and modulates tau phosphorylation.* Ann Neurol, 2014. **76**(3): p. 379-92.

14. Lambert, J.C., et al., *Meta-analysis of 74,046 individuals identifies 11 new susceptibility loci for Alzheimer's disease.* Nat Genet, 2013. **45**(12): p. 1452-8.

15. Gerrish, A., et al., *The role of variation at AβPP, PSEN1, PSEN2, and MAPT in late onset Alzheimer's disease.* Journal of Alzheimer's disease : JAD, 2012. **28**(2): p. 377-387.

16. Reiman, E.M., et al., *GAB2 alleles modify Alzheimer's risk in APOE epsilon4 carriers.* Neuron, 2007. **54**(5): p. 713-20.

17. Bernhardt, B.C., et al., *Longitudinal and cross-sectional analysis of atrophy in pharmacoresistant temporal lobe epilepsy.* Neurology, 2009. **72**(20): p. 1747-1754.

18. Giau, V.V., et al., *Analysis of 50 Neurodegenerative Genes in Clinically Diagnosed Early-Onset Alzheimer's Disease.* International journal of molecular sciences, 2019. **20**(6): p. 1514.

19. Liu, J., et al., *Structural Insights into SHARPIN-Mediated Activation of HOIP for the Linear Ubiquitin Chain Assembly.* Cell Reports, 2017. **21**(1): p. 27-36.

20. Pettersen, E.F., et al., *UCSF Chimera—A visualization system for exploratory research and analysis.* Journal of Computational Chemistry, 2004. **25**(13): p. 1605-1612.

21. Jo, S., et al., *CHARMM‐GUI: a web‐based graphical user interface for CHARMM.* 2008. **29**(11): p. 1859-1865.

22. DeLano, W.L., *PyMOL*. 2002.

23. Vanommeslaeghe, K., et al., *CHARMM general force field: A force field for drug‐like molecules compatible with the CHARMM all‐atom additive biological force fields.* 2010. **31**(4): p. 671-690.

24. Phillips, J.C., et al., *Scalable molecular dynamics with NAMD.* 2005. **26**(16): p. 1781-1802.

25. Humphrey, W., A. Dalke, and K.J.J.o.m.g. Schulten, *VMD: visual molecular dynamics.* 1996. **14**(1): p. 33-38.

26. Grant, B.J., et al., *Bio3d: an R package for the comparative analysis of protein structures.* 2006. **22**(21): p. 2695-2696.
