# Supplementary material for: A missense variant in SHARPIN mediates Alzheimer’s disease-specific brain damages
Source: Transl Psychiatry. 2021 Nov 16;11:590. doi: 10.1038/s41398-021-01680-5 (PMC8595886; doi:10.1038/s41398-021-01680-5)
Supplement: Supplementary file 2 — Supplementary Figure legends and Table tiles [file 41398_2021_1680_MOESM2_ESM.docx]

**Supplementary Figure 1. Analysis flow chart.** Quality-control (QC) filtering steps for selection of SNPs and subjects included in GWAS. AD: Alzheimer disease cases; MCI: mild cognitive impairment cases; CN: cognitively normal subjects.

**Supplementary Figure 2.** **Population substructure analysis of the GWAS discovery sample.** Population substructure was evaluated by analysis of principle components (PCs) of ancestry. Plots of pairwise comparisons for two of the first three PCs generated from the Korean GWAS discovery sample (red dots) and 1,000 Genomes reference panel (grey dots) show little overlap between the Korean GWAS sample and 1,000 Genome subjects who are primarily of European ancestry and African Americans.

**Supplementary Figure 3. Manhattan plots illustrating results of the *APOE* genotype-adjusted GWAS for five MRI traits.** Red lines indicate the genome-wide significance threshold (p=5.0x10^-8^) and blue lines indicate the threshold for suggestive evidence for association (p=1.0x10^-5^).

**Supplementary Figure 4.** **Quintile-quintile (QQ) plots of GWAS for five MRI traits.** They show little evidence of genomic inflation; 𝛌: measure of genomic inflation.

**Supplementary Figure 5. Results of genome-wide gene-based analysis with rare variants for hippocampal volume and entorhinal cortical thickness.** (**A**) Manhattan plots show results for each gene. Blue line indicates the genome-wide significance threshold (p=2.6x10^-6^). **(B)** QQ plots show little evidence of genomic inflation for hippocampal volume (𝛌=0.992) and entorhinal thickness (𝛌=1.023).

**Supplementary Figure 6. Rotated and zoomed views for the structural changes at HOIP^UBA^-SHARPIN^UBL^ complex in MD simulations.** The structural differences at the interface between WT and mutant complexes are rotated and zoomed in to provide clear view in (A), (B) and (C). The left panels show the conformational change in the structural element level and the right panels show the residues that contribute to the conformational change. The deviations at the structural element and residue levels are marked by arrow and the distance indicated in Å.

**Supplementary Figure 7. Surface model of the complex colored based on electrostatic potential.** Electrostatic surface view of the HOIP^UBA^-SHARPIN^UBL^ complex in (**A**) WT and (**B**) mutant, from the structures obtained after averaging the last 20 ns of the total 60 ns simulation, is colored by the local electrostatic potential (blue, +2 kT; red −2 kT), respectively. We could clearly see the change in charge at the interface where Arg274 is present (boxed) between WT and mutant.

**Supplementary Figure 8. Surface model colored based on hydrophobicity.** Hydrophobic Surface model of HOIP^UBA^-SHARPIN^UBL^ for (**A**) WT and (**B**) mutant after 60 ns simulation, respectively, colored based on hydrophobicity (increasing white to red). The increase in hydrophobicity correlates to the red patch proximal to mutant tryptophan (boxed) and the corresponding residues that contribute to the hydrophobicity is zoomed in with a box

**Supplementary Table** **1. Characteristics of the GWAS discovery sample.**

| **Traits** | **Mean ± [SD]** | | | | **ANOVA** |
| --- | --- | --- | --- | --- | --- |
|  | **Total** | **AD** | **MCI** | **CN** | **(P)** |
| N | 2643 | 209 | 1449 | 985 | - |
| Age (year) | $73[5.8]$ | $73.7[7.4]$ | $73.2[5.8]$ | $72.5[5.4]$ | $0.001$ |
| Sex(M/F) | $1071/1572$ | $88/121$ | $605/844$ | $378/607$ | $0.222$ |
| Entorhinal (mm) | $6.8[0.8]$ | $5.8[1.1]$ | $6.8[0.7]$ | $7[0.6]$ | $<0.001$ |
| Inferior parietal (mm) | $4.5[0.4]$ | $4.3[0.4]$ | $4.5[0.4]$ | $4.6[0.3]$ | $<0.001$ |
| Middle temporal (mm) | $5.3[0.4]$ | $5[0.6]$ | $5.3[0.4]$ | $5.4[0.3]$ | $<0.001$ |
| Superior frontal (mm) | $5.1[0.3]$ | $4.9[0.4]$ | $5.1[0.3]$ | $5.1[0.3]$ | $<0.001$ |
| Hippocampus (mm^3^) | $7533[1096]$ | $6254[1166.1]$ | $7500[1023.4]$ | $7853[972.2]$ | $<0.001$ |

**Supplementary Table 2. Descriptive statistics by Aβ status.**

| Variable | Level | Statistics | Total  (N = 1,377) | Positive  (N = 418) | Negative  (N = 959) |
| --- | --- | --- | --- | --- | --- |
| Age |  | mean[sd] | 72.2[6.1] | 73.1[6.4] | 71.9[5.9] |
| Sex |  |  |  |  |  |
|  | M | N(%) | 686(50) | 232(55) | 454(47) |
|  | F | N(%) | 691(50) | 186(45) | 505(53) |
|  | Total | N(%) | 1,377(100) | 418(100) | 959(100) |
| AD status |  |  |  |  |  |
|  | CN | N(%) | 628(46) | 134(32) | 494(52) |
|  | MCI | N(%) | 587(43) | 165(50) | 422(44) |
|  | AD | N(%) | 162(11) | 119(28) | 43(4) |
|  | Total | N(%) | 1,377(100) | 418(100) | 959(100) |

**Supplementary Table 3. Association of *APOE* genotype with MRI traits**

| **Trait** | **Genotypes** | **GF** | **stat.** | **dist.** | $\boldsymbol{\beta}$ | **SE** | **P value** |
| --- | --- | --- | --- | --- | --- | --- | --- |
| Entorhinal thickness | - | - | LRT | $\chi^{2}(DF=5)$ | - | - | $5.1\times{10}^{-11}$ |
|  | ε4ε4 | 0.014 | Wald | Z | -1.05 | 0.16 | $5.7\times{10}^{-11}$ |
|  | ε3ε4 | 0.183 | Wald | Z | -0.17 | 0.04 | $3.0\times{10}^{-4}$ |
|  | ε2ε4 | 0.012 | Wald | Z | 0.01 | 0.17 | $0.95$ |
|  | ε2ε3 | 0.100 | Wald | Z | 0.06 | 0.06 | $0.27$ |
|  | ε2ε2 | 0.004 | Wald | Z | -0.02 | 0.30 | $0.93$ |
|  | ε3ε3 | 0.687 | reference | - | - | - | - |
| Inferior parietal thickness | - | - | LRT | $\chi^{2}(DF=5)$ | - | - | $4.2\times{10}^{-8}$ |
|  | ε4ε4 | 0.014 | Wald | Z | -0.65 | 0.16 | $5.2\times{10}^{-5}$ |
|  | ε3ε4 | 0.183 | Wald | Z | -0.25 | 0.05 | $3.4\times{10}^{-7}$ |
|  | ε2ε4 | 0.012 | Wald | Z | 0.15 | 0.17 | 0.37 |
|  | ε2ε3 | 0.100 | Wald | Z | -0.05 | 0.06 | 0.41 |
|  | ε2ε2 | 0.004 | Wald | Z | 0.27 | 0.30 | 0.37 |
|  | ε3ε3 | 0.687 | reference | - | - | - | - |
| Middle temporal thickness | - | - | LRT | $\chi^{2}(DF=5)$ | - | - | $5.8\times{10}^{-7}$ |
|  | ε4ε4 | 0.014 | Wald | Z | -0.65 | 0.16 | $4.1\times{10}^{-5}$ |
|  | ε3ε4 | 0.183 | Wald | Z | -0.21 | 0.05 | $1.3\times{10}^{-5}$ |
|  | ε2ε4 | 0.012 | Wald | Z | 0.20 | 0.17 | 0.23 |
|  | ε2ε3 | 0.100 | Wald | Z | 0.01 | 0.06 | 0.89 |
|  | ε2ε2 | 0.004 | Wald | Z | 0.07 | 0.30 | 0.28 |
|  | ε3ε3 | 0.687 | reference | - | - | - | - |
| Superior frontal thickness | - | - | LRT | $\chi^{2}(DF=5)$ | - | - | $3.9\times{10}^{-8}$ |
|  | ε4ε4 | 0.014 | Wald | Z | -0.55 | 0.16 | $7.0\times{10}^{-4}$ |
|  | ε3ε4 | 0.183 | Wald | Z | -0.28 | 0.05 | $1.7\times{10}^{-8}$ |
|  | ε2ε4 | 0.012 | Wald | Z | 0.09 | 0.17 | 0.59 |
|  | ε2ε3 | 0.100 | Wald | Z | -0.12 | 0.06 | 0.06 |
|  | ε2ε2 | 0.004 | Wald | Z | 0.07 | 0.30 | 0.83 |
|  | ε3ε3 | 0.687 | reference | - | - | - | - |
| hippocampal volume | - | - | LRT | $\chi^{2}(DF=5)$ | - | - | $6.3\times{10}^{-20}$ |
|  | ε4ε4 | 0.014 | Wald | Z | -1.22 | 0.14 | $9.4\times{10}^{-18}$ |
|  | ε3ε4 | 0.183 | Wald | Z | -0.21 | 0.04 | $4.5\times{10}^{-7}$ |
|  | ε2ε4 | 0.012 | Wald | Z | 0.08 | 0.15 | $0.55$ |
|  | ε2ε3 | 0.100 | Wald | Z | 0.05 | 0.05 | $0.33$ |
|  | ε2ε2 | 0.004 | Wald | Z | 0.14 | 0.26 | $0.58$ |
|  | ε3ε3 | 0.687 | reference | - | - | - | - |

GF: genotype frequency, LRT: likelihood ratio test

**Supplementary Table 4. Significant GWAS Results (p<1.0x10^-6^) for MRI traits unadjusted for *APOE* genotype**

| **Trait** | **Chr** | **Position** | **SNP** | **MA** | **MAF** | **IQS** | $\boldsymbol{\beta}$ | **SE** | **P value** | **Locus** |
| --- | --- | --- | --- | --- | --- | --- | --- | --- | --- | --- |
| Entorhinal thickness | 19 | 45411941 | rs429358 | C | 0.11 | G | -0.25 | 0.04 | $1.1\times{10}^{-9}$ | *APOE* |
|  | 8 | 145154282 | rs77359862 | A | 0.02 | G | -0.58 | 0.10 | $1.9\times{10}^{-8}$ | *SHARPIN* |
|  | 19 | 45422160 | rs12721051 | G | 0.12 | 0.99 | -0.23 | 0.04 | $2.1\times{10}^{-8}$ | *APOC1* |
|  | 19 | 45422846 | rs56131196 | A | 0.12 | 0.99 | -0.23 | 0.04 | $2.9\times{10}^{-8}$ | *APOC1* |
|  | 19 | 45422946 | rs4420638 | G | 0.12 | G | -0.22 | 0.04 | $7.9\times{10}^{-8}$ | *APOC1* |
|  | 19 | 45396144 | rs11556505 | T | 0.11 | 0.99 | -0.23 | 0.04 | $1.4\times{10}^{-7}$ | *TOMM40* |
|  | 19 | 45392254 | rs6857 | T | 0.10 | 0.99 | -0.23 | 0.04 | $1.4\times{10}^{-7}$ | *NECTIN2* |
|  | 19 | 45410002 | rs769449 | A | 0.09 | G | -0.25 | 0.05 | $1.6\times{10}^{-7}$ | *APOE* |
|  | 19 | 45395909 | rs34404554 | G | 0.11 | 0.99 | -0.23 | 0.04 | $2.1\times{10}^{-7}$ | *TOMM40* |
|  | 19 | 45394336 | rs71352238 | C | 0.11 | I.99 | -0.22 | 0.04 | $3.0\times{10}^{-7}$ | *TOMM40* |
|  | 19 | 45395619 | rs2075650 | G | 0.11 | G | -0.22 | 0.04 | $3.1\times{10}^{-7}$ | *TOMM40* |
|  | 19 | 45388130 | rs34342646 | A | 0.10 | G | -0.22 | 0.04 | $3.3\times{10}^{-7}$ | *NECTIN2* |
|  | 18 | 59237056 | rs147834038 | T | 0.03 | G | -0.41 | 0.08 | $4.5\times{10}^{-7}$ | *CDH20* |
|  | 14 | 27219914 | rs1956822 | G | 0.39 | 0.99 | -0.14 | 0.03 | $5.0\times{10}^{-7}$ | *NOVA1-AS1* |
|  | 19 | 45387459 | rs12972156 | G | 0.10 | 0.99 | -0.22 | 0.04 | $5.2\times{10}^{-7}$ | *NECTIN2* |
|  | 19 | 45387596 | rs12972970 | A | 0.10 | 0.99 | -0.22 | 0.04 | $5.2\times{10}^{-7}$ | *NECTIN2* |
|  | 14 | 27221601 | rs7160806 | G | 0.39 | 0.99 | -0.14 | 0.03 | $6.3\times{10}^{-7}$ | *NOVA1-AS1* |
|  | 14 | 27219292 | rs8006420 | A | 0.39 | 0.99 | -0.13 | 0.03 | $7.5\times{10}^{-7}$ | *NOVA1-AS1* |
|  | 14 | 27219302 | rs8006426 | G | 0.39 | 0.99 | -0.13 | 0.03 | $7.5\times{10}^{-7}$ | *NOVA1-AS1* |
|  | 14 | 27218970 | rs10150868 | T | 0.39 | G | -0.13 | 0.03 | $7.8\times{10}^{-7}$ | *NOVA1-AS1* |
|  | 14 | 27219237 | rs8021452 | T | 0.39 | 0.99 | -0.13 | 0.03 | $8.0\times{10}^{-7}$ | *NOVA1-AS1* |
|  | 14 | 27223432 | rs11847395 | T | 0.39 | 0.99 | -0.13 | 0.03 | $9.1\times{10}^{-7}$ | *NOVA1-AS1* |
|  | 14 | 27220620 | rs8022789 | T | 0.39 | 0.99 | -0.13 | 0.03 | $9.6\times{10}^{-7}$ | *NOVA1-AS1* |
|  | 14 | 27221318 | rs7145094 | T | 0.39 | 0.99 | -0.13 | 0.03 | $9.6\times{10}^{-7}$ | *NOVA1-AS1* |
|  | 14 | 27222267 | rs17111690 | G | 0.39 | 0.99 | -0.13 | 0.03 | $9.6\times{10}^{-7}$ | *NOVA1-AS1* |
| Inferior parietal thickness | 19 | 45422846 | rs56131196 | A | 0.12 | 0.99 | -0.24 | 0.04 | $5.1\times{10}^{-9}$ | *APOC1* |
|  | 19 | 45411941 | rs429358 | C | 0.11 | G | -0.24 | 0.04 | $5.3\times{10}^{-9}$ | *APOE* |
|  | 19 | 45422160 | rs12721051 | G | 0.12 | 0.99 | -0.24 | 0.04 | $6.1\times{10}^{-9}$ | *APOC1* |
|  | 19 | 45422946 | rs4420638 | G | 0.12 | G | -0.23 | 0.04 | $1.3\times{10}^{-8}$ | *APOC1* |
|  | 19 | 45415713 | rs10414043 | A | 0.09 | 0.99 | -0.24 | 0.05 | $2.5\times{10}^{-7}$ | *APOE / APOC1* |
|  | 19 | 45410002 | rs769449 | A | 0.09 | G | -0.24 | 0.05 | $4.3\times{10}^{-7}$ | *APOE* |
|  | 19 | 45387459 | rs12972156 | G | 0.10 | 0.99 | -0.22 | 0.04 | $5.0\times{10}^{-7}$ | *NECTIN2* |
|  | 19 | 45387596 | rs12972970 | A | 0.10 | 0.99 | -0.22 | 0.04 | $5.0\times{10}^{-7}$ | *NECTIN2* |
|  | 19 | 45388130 | rs34342646 | A | 0.10 | G | -0.22 | 0.04 | $5.2\times{10}^{-7}$ | *NECTIN2* |
|  | 19 | 45415935 | rs7256200 | T | 0.09 | 0.99 | -0.23 | 0.05 | $5.4\times{10}^{-7}$ | *APOE / APOC1* |
|  | 19 | 45392254 | rs6857 | T | 0.10 | 0.99 | -0.22 | 0.04 | $5.8\times{10}^{-7}$ | *NECTIN2* |
| Middle temporal thickness | 19 | 45422160 | rs12721051 | G | 0.12 | 0.99 | -0.22 | 0.04 | $5.1\times{10}^{-8}$ | *APOC1* |
|  | 19 | 45422846 | rs56131196 | A | 0.12 | 0.99 | -0.21 | 0.04 | $7.9\times{10}^{-8}$ | *APOC1* |
|  | 19 | 45422946 | rs4420638 | G | 0.12 | G | -0.20 | 0.04 | $2.6\times{10}^{-7}$ | *APOC1* |
|  | 19 | 45411941 | rs429358 | C | 0.11 | G | -0.21 | 0.04 | $3.2\times{10}^{-7}$ | *APOE* |
|  | 19 | 45415713 | rs10414043 | A | 0.09 | 0.99 | -0.23 | 0.05 | $5.2\times{10}^{-7}$ | *APOE / APOC1* |
|  | 19 | 45392254 | rs6857 | T | 0.10 | 0.99 | -0.21 | 0.04 | $8.2\times{10}^{-7}$ | *NECTIN2* |
|  | 19 | 45410002 | rs769449 | A | 0.09 | G | -0.23 | 0.05 | $9.5\times{10}^{-7}$ | *APOE* |
| Superior frontal thickness | 19 | 45411941 | rs429358 | C | 0.11 | G | -0.24 | 0.04 | $1.3\times{10}^{-8}$ | *APOE* |
|  | 19 | 45422160 | rs12721051 | G | 0.12 | 0.99 | -0.23 | 0.04 | $1.9\times{10}^{-8}$ | *APOC1* |
|  | 19 | 45422846 | rs56131196 | A | 0.12 | 0.99 | -0.23 | 0.04 | $2.6\times{10}^{-8}$ | *APOC1* |
|  | 19 | 45422946 | rs4420638 | G | 0.12 | G | -0.22 | 0.04 | $7.9\times{10}^{-8}$ | *APOC1* |
|  | 19 | 45410002 | rs769449 | A | 0.09 | G | -0.26 | 0.05 | $1.3\times{10}^{-7}$ | *APOE* |
|  | 19 | 45415713 | rs10414043 | A | 0.09 | 0.99 | -0.24 | 0.05 | $2.7\times{10}^{-7}$ | *APOE / APOC1* |
|  | 19 | 45416741 | rs438811 | T | 0.18 | 0.99 | -0.18 | 0.04 | $4.2\times{10}^{-7}$ | *APOC1* |
|  | 19 | 45416178 | rs483082 | T | 0.18 | 0.99 | -0.18 | 0.04 | $4.2\times{10}^{-7}$ | *APOE / APOC1* |
|  | 19 | 45415935 | rs7256200 | T | 0.09 | 0.99 | -0.23 | 0.05 | $7.9\times{10}^{-7}$ | *APOE / APOC1* |
| Hippo-campal volume | 19 | 45411941 | rs429358 | C | 0.11 | G | -0.30 | 0.04 | $1.1\times{10}^{-15}$ | *APOE* |
|  | 19 | 45422846 | rs56131196 | A | 0.12 | 0.99 | -0.27 | 0.04 | $5.9\times{10}^{-14}$ | *APOC1* |
|  | 19 | 45422160 | rs12721051 | G | 0.12 | 0.99 | -0.27 | 0.04 | $2.6\times{10}^{-13}$ | *APOC1* |
|  | 19 | 45422946 | rs4420638 | G | 0.12 | G | -0.26 | 0.04 | $7.0\times{10}^{-13}$ | *APOC1* |
|  | 19 | 45410002 | rs769449 | A | 0.09 | G | -0.30 | 0.04 | $1.0\times{10}^{-12}$ | *APOE* |
|  | 19 | 45392254 | rs6857 | T | 0.10 | 0.99 | -0.27 | 0.04 | $6.0\times{10}^{-12}$ | *NECTIN2* |
|  | 19 | 45388130 | rs34342646 | A | 0.10 | G | -0.26 | 0.04 | $1.3\times{10}^{-11}$ | *NECTIN2* |
|  | 19 | 45396144 | rs11556505 | T | 0.11 | 0.99 | -0.26 | 0.04 | $1.4\times{10}^{-11}$ | *TOMM40* |
|  | 19 | 45394336 | rs71352238 | C | 0.11 | 0.99 | -0.26 | 0.04 | $1.7\times{10}^{-11}$ | *TOMM40* |
|  | 19 | 45395909 | rs34404554 | G | 0.11 | 0.99 | -0.26 | 0.04 | $1.8\times{10}^{-11}$ | *TOMM40* |
|  | 19 | 45395619 | rs2075650 | G | 0.11 | G | -0.26 | 0.04 | $1.8\times{10}^{-11}$ | *TOMM40* |
|  | 19 | 45387459 | rs12972156 | G | 0.10 | 0.99 | -0.26 | 0.04 | $2.6\times{10}^{-11}$ | *NECTIN2* |
|  | 19 | 45387596 | rs12972970 | A | 0.10 | 0.99 | -0.26 | 0.04 | $2.6\times{10}^{-11}$ | *NECTIN2* |
|  | 8 | 145154282 | rs77359862 | A | 0.02 | G | -0.60 | 0.09 | $5.4\times{10}^{-11}$ | *SHARPIN* |
|  | 19 | 45415713 | rs10414043 | A | 0.09 | 0.99 | -0.25 | 0.04 | $4.4\times{10}^{-10}$ | *APOE / APOC1* |
|  | 19 | 45415935 | rs7256200 | T | 0.09 | 0.99 | -0.25 | 0.04 | $1.0\times{10}^{-9}$ | *APOE / APOC1* |
|  | 8 | 144984345 | rs80120848 | A | 0.02 | G | -0.51 | 0.04 | $1.8\times{10}^{-7}$ | *EPPK1/PLEC* |

Chr: chromosome; MA: minor allele; MAF: minor allele frequency; IQS: imputation quality score; G: genotyped SNP; SE: standard error

**Supplementary Table 5. GWAS Results for previously reported AD loci.**

| **Gene** | **Chr** | **Position** | **SNP** | **Replacement SNP** | $\boldsymbol{D}^{\boldsymbol{'}}$ | $\boldsymbol{r}^{\boldsymbol{2}}$ | **MA** | $\boldsymbol{\beta}$ | ***p*-value** |
| --- | --- | --- | --- | --- | --- | --- | --- | --- | --- |
| *TREM2* | 6 | 41129252 | rs75932628 |  |  |  | T | -0.65 | 0.28 |
| *BIN1* | 2 | 127892810 | rs6733839 | rs4663105 | 0.972 | 0.743 | C | -0.05 | 0.04 |
| *CLU* | 8 | 27467686 | rs9331896 |  |  |  | C | 0.04 | 0.09 |
| *PICALM* | 11 | 85867875 | rs10792832 |  |  |  | A | 0.01 | 0.60 |
| *INPP5D* | 2 | 234068476 | rs35349669 |  |  |  | T | -0.01 | 0.89 |
| *MS4A6A* | 11 | 59923508 | rs983392 | rs7124974 | 1.0 | 0.979 | T | -0.01 | 0.88 |
| *CR1* | 1 | 207692049 | rs6656401 |  |  |  | A | -0.01 | 0.88 |
| *HLA* | 6 | 32578530 | rs9271192 |  |  |  | C | -0.03 | 0.39 |
| *ZCWPW1* | 7 | 100004446 | rs1476679 |  |  |  | C | 0.005 | 0.83 |
| *EPHA1* | 7 | 143110762 | rs11771145 |  |  |  | A | -0.003 | 0.92 |
| *PTK2B* | 8 | 27195121 | rs28834970 | rs6987305 | 0.995 | 0.990 | A | 0.002 | 0.95 |
| *NME8* | 7 | 37841534 | rs2718058 |  |  |  | G | 0.04 | 0.19 |
| *ABCA7* | 19 | 1063443 | rs4147929 | rs3752246 | 0.983 | 0.961 | G | -0.10 | $3.9 \times{10}^{-5}$ |
| *CELF1* | 11 | 47557871 | rs10838725 |  |  |  | C | -0.05 | 0.04 |
| *DSG2* | 6 | 29088958 | rs8093731 |  |  |  | T | -0.06 | 0.24 |
| *FERMT2* | 14 | 53400629 | rs17125944 |  |  |  | C | -0.02 | 0.42 |
| *PLXNA4* | 7 | 132037683 | rs12539196 |  |  |  | C | 0.05 | 0.61 |
| *FERMT2* | 14 | 53400629 | rs17125944 |  |  |  | C | -0.02 | 0.45 |
| *SORL1* | 11 | 121435587 | rs11218343 |  |  |  | C | 0.02 | 0.40 |
| *CD2AP* | 6 | 47487762 | rs10948363 |  |  |  | G | -0.02 | 0.45 |
| *CD33* | 19 | 51727962 | rs3865444 |  |  |  | A | 0.01 | 0.61 |
| *GAB2* | 11 | 77936166 | rs1385600 |  |  |  | G | 0.05 | 0.04 |
| *PSEN1* | 14 | 73631092 | rs362350 |  |  |  | G | 0.18 | 0.06 |

Chr: chromosome; MA: minor allele

**Supplementary Table 6. Characteristics of prospectively followed subjects**

|  | Total | stable | converters |
| --- | --- | --- | --- |
| N | 876 | 802 | 74 |
| Age, mean ± sd | 73.9 ± 4.8 | 73.7 ± 4.7 | 75.3 ± 5.1 |
| Female, N (%) | 384 (43.8%) | 351 (43.8%) | 33 (44.6%) |
| rs77359862 mutant allele, N (%) | 32 (3.7%) | 26 (3.2%) | 6 (8.1%) |
| APOE ε4 carrier, N (%) | 214 (24.4%) | 190 (23.7%) | 24 (32.4%) |
| Follow-up period (months), mean± sd | 28.8 ± 15.8 | 28.5 ± 15.9 | 31.8 ± 15.8 |

* Age: age at AD onset.

**Supplementary Table 7. Number of hydrogen bonds and salt bridges at the interface of HOIP^UBA^-SHARPIN^UBL^ complex in (A) WT and (B) mutant.**

**(A) HOIP^UBA^-SHARPIN^UBL^ complex (WT)**

Salt bridges

| **No.** | **HOIP^UBA^** | **Distance (Å)** | **SHARPIN^UBL^** |
| --- | --- | --- | --- |
| 1 | A:GLU 487[ OE1] | 2.73 | B:ARG 274[ NH2] |
| 2 | A:GLU 487[ OE2] | 3.50 | B:ARG 274[ NH2] |
| 3 | A:GLU 506[ OE1] | 2.80 | B:ARG 269[ NH2] |
| 4 | A:GLU 506[ OE2] | 2.76 | B:ARG 269[ NE ] |
| 5 | A:GLU 506[ OE2] | 3.28 | B:ARG 269[ NH2] |
| 6 | A:ARG 496[ NH1] | 3.36 | B:GLU 226[ OE1] |
| 7 | A:ARG 496[ NH1] | 2.61 | B:GLU 226[ OE2] |

Hydrogen bonds

| **No.** | **HOIP^UBA^** | **Distance (Å)** | **SHARPIN^UBL^** |
| --- | --- | --- | --- |
| 1 | A:GLU 499 [ OE1] | 2.89 | B:SER 301 [ N  ] |
| 2 | A:GLU 499 [ OE2] | 2.68 | B:SER 301 [ OG ] |
| 3 | A:GLN 490 [ OE1] | 2.69 | B:ARG 274 [ NH2] |
| 4 | A:GLN 481 [ NE2] | 2.80 | B:ASP 293 [ OD1] |
| 5 | A:ARG 485 [ NH1] | 3.08 | B:PRO 294 [ O ] |
| 6 | A:ARG 485 [ NH2] | 2.65 | B:PRO 294 [ O ] |
| 7 | A:GLU 506 [ OE2] | 2.78 | B:ARG 269 [ NE1] |
| 8 | A:ARG 496 [ OE1] | 3.16 | B:SER 230 [ N ] |
| 9 | A:GLU 487 [ OE1] | 3.55 | B:ARG 274 [ NE ] |
| 10 | A:GLU 487 [ OE2] | 2.77 | B:ARG 274 [ NE ] |

**(B)** **HOIP^UBA^-SHARPIN^UBL^ complex (R274W)**

Hydrogen bonds

| \| **No.** \| **HOIP^UBA^** \| **Distance (Å)** \| **SHARPIN^UBL^** \| \| --- \| --- \| --- \| --- \| \| 1 \| A:GLN 481[ NE2] \| 3.60 \| B:ASP 293[ OD2] \| \| 2 \| A:ARG 496[ NH1] \| 3.48 \| B:SER 230[ O  ] \| \| 3 \| A:ARG 496[ NH1] \| 3.66 \| B:ALA 228[ O  ] \| \| 4 \| A:ARG 496[ NH2] \| 3.25 \| B:SER 230[ O  ] \| |
| --- | --- | --- | --- | --- | --- | --- | --- | --- | --- | --- | --- | --- | --- | --- | --- | --- | --- | --- | --- | --- |
