# Supplementary figures and images for: A missense variant in SHARPIN mediates Alzheimer’s disease-specific brain damages
Source: Transl Psychiatry. 2021 Nov 16;11:590. doi: 10.1038/s41398-021-01680-5 (PMC8595886; doi:10.1038/s41398-021-01680-5)

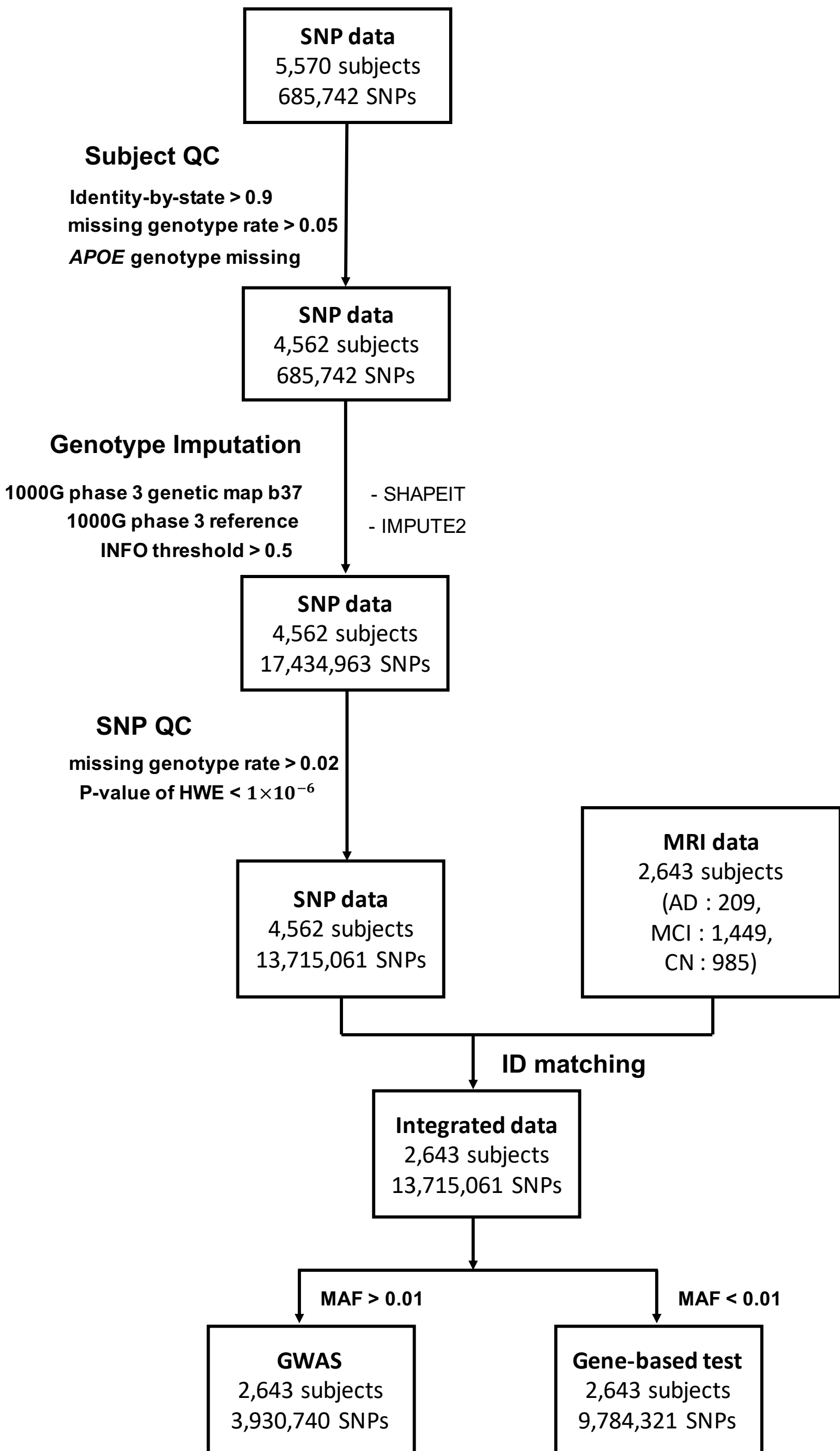

Supplement: Supplementary file 3 — Supplemental Figure1 [file 41398_2021_1680_MOESM3_ESM.pdf]

**(A) PC1 vs PC2**

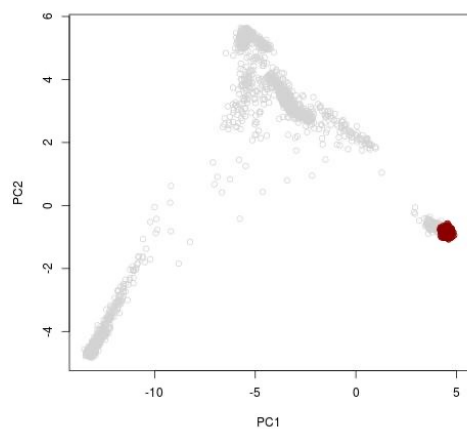

**(B) PC2 vs PC3**

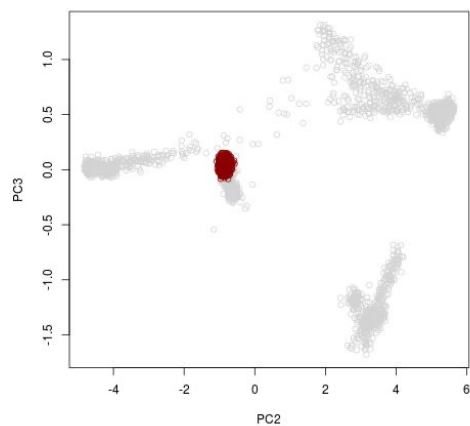

**(B) PC1 vs PC3**

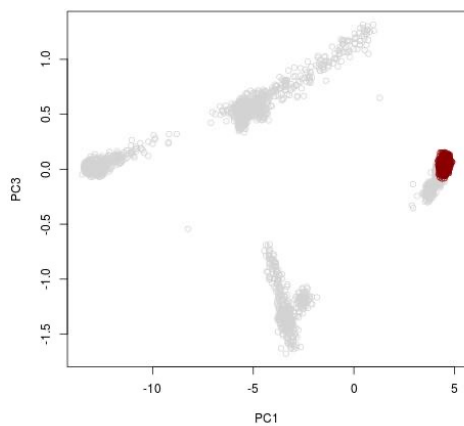

Supplement: Supplementary file 4 — Supplemental Figure2 [file 41398_2021_1680_MOESM4_ESM.pdf]

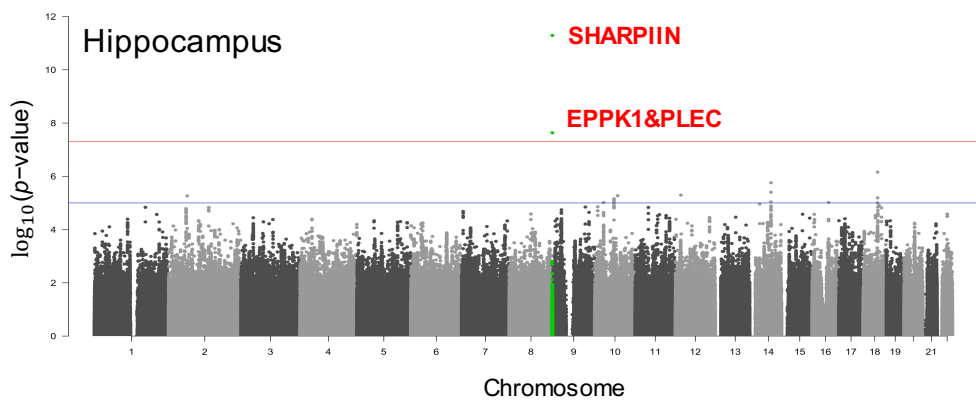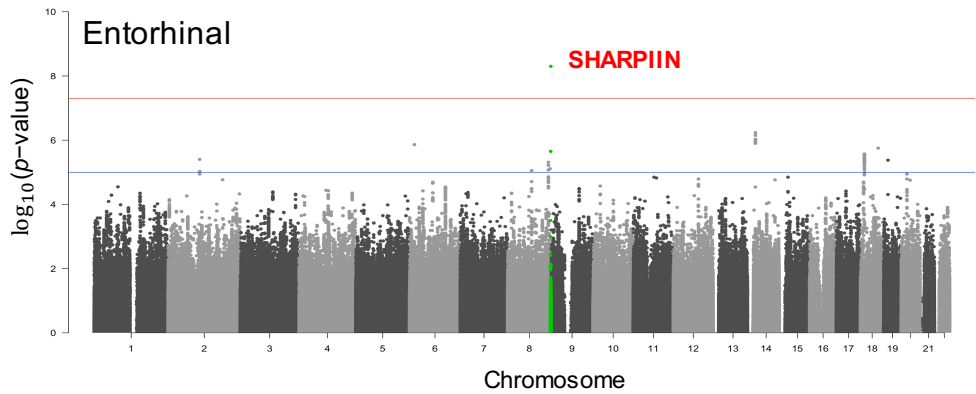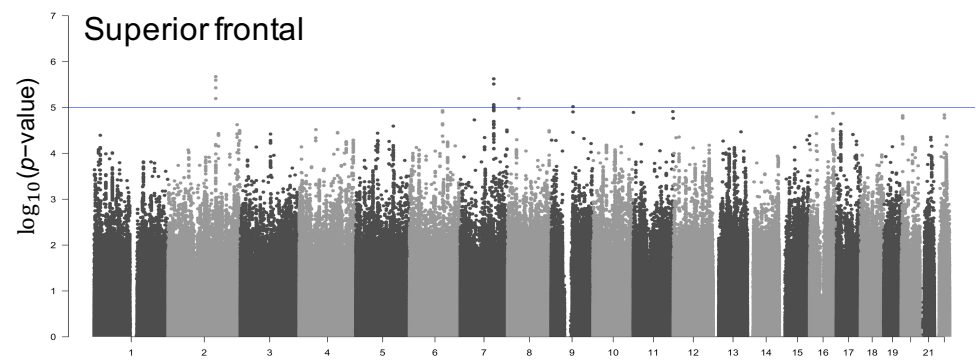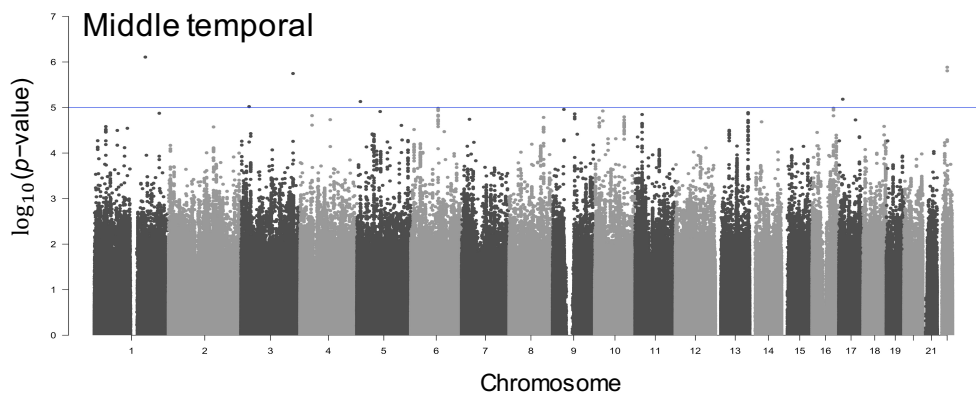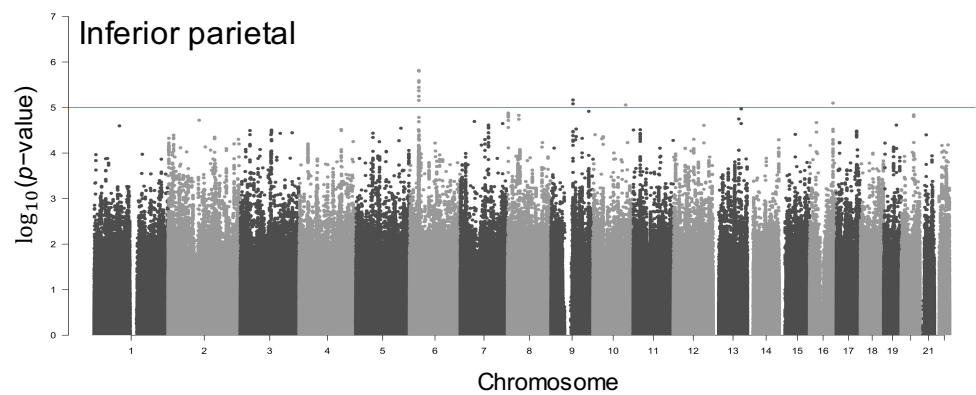

Supplement: Supplementary file 5 — Supplemental Figure3 [file 41398_2021_1680_MOESM5_ESM.pdf]

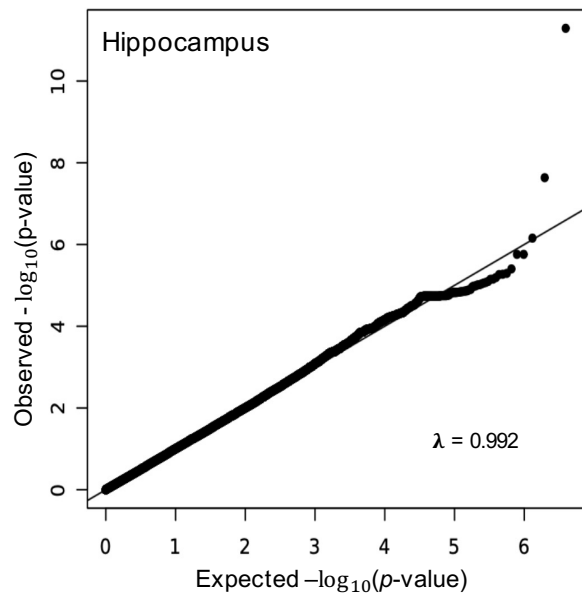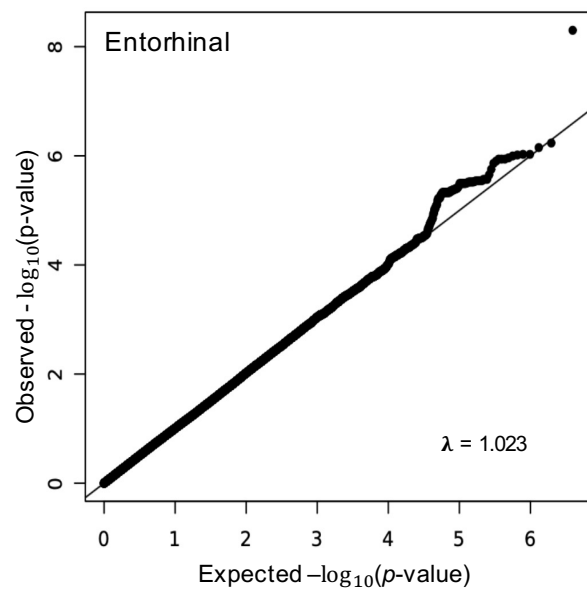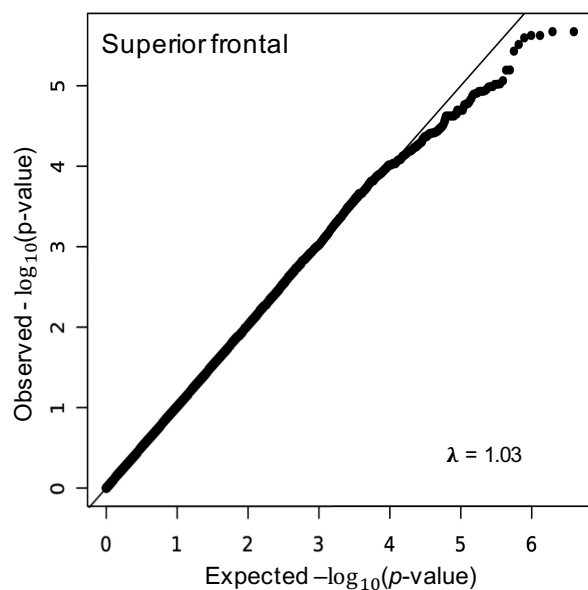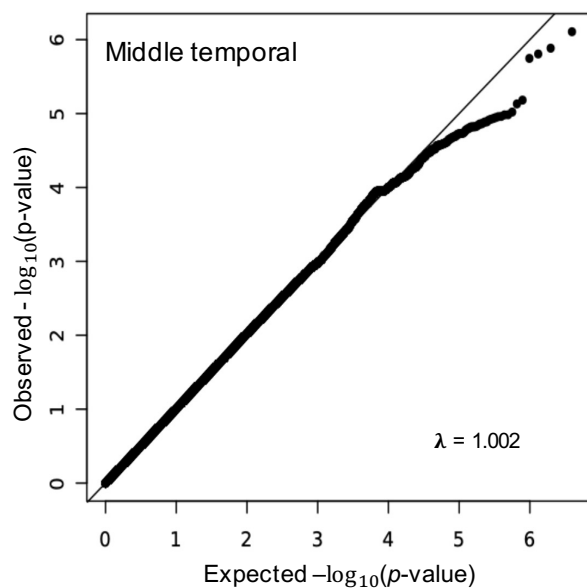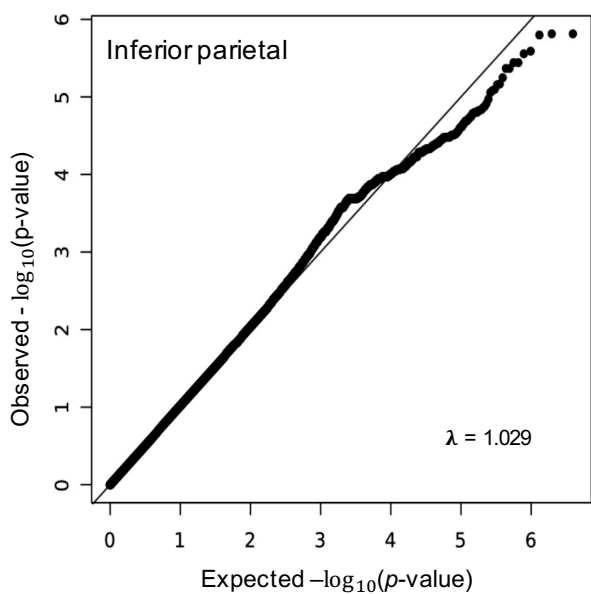

Supplement: Supplementary file 6 — Supplemental Figure4 [file 41398_2021_1680_MOESM6_ESM.pdf]

(A)

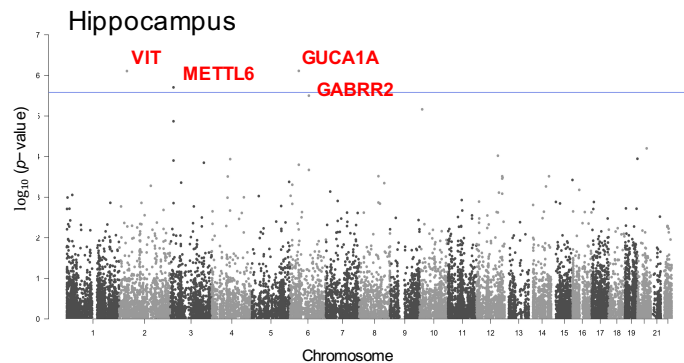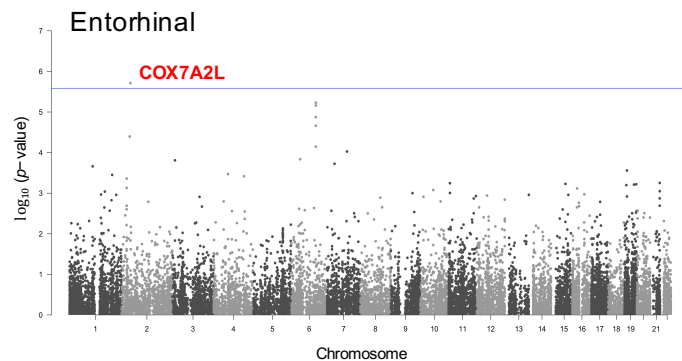

(B)

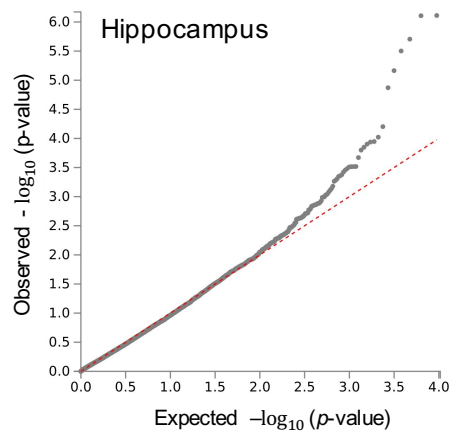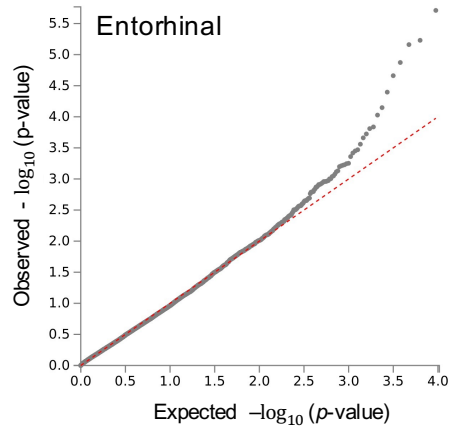

Supplement: Supplementary file 7 — Supplemental Figure5 [file 41398_2021_1680_MOESM7_ESM.pdf]

(A)

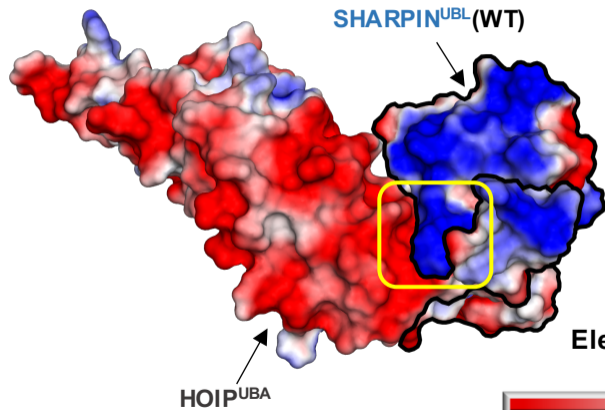

(B)

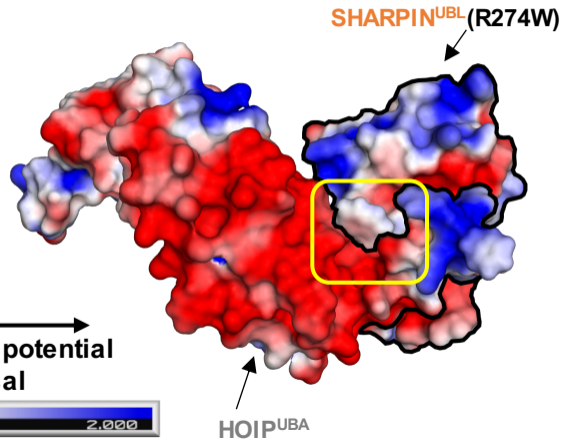

Electrostatic potential  
reversal

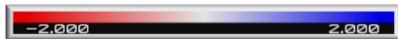

Supplement: Supplementary file 9 — Supplemental Figure7 [file 41398_2021_1680_MOESM9_ESM.pdf]

(A)

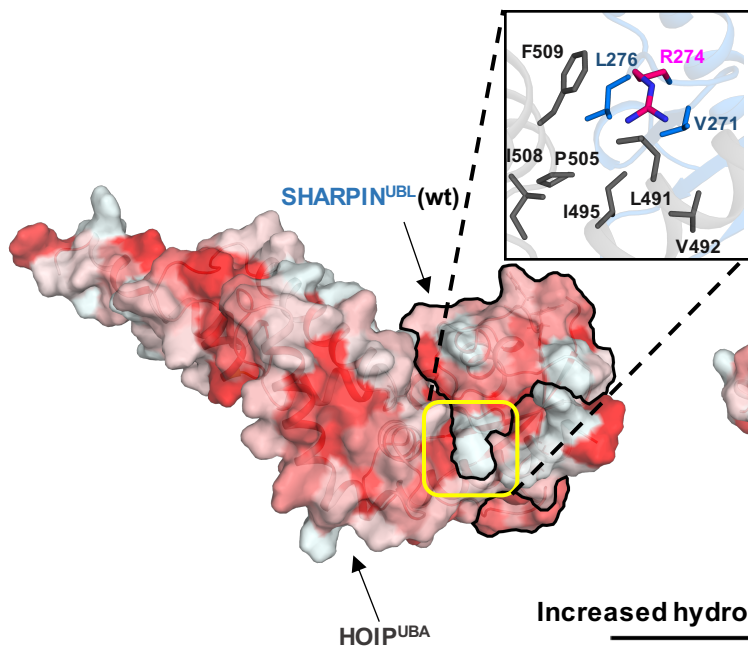

(B)

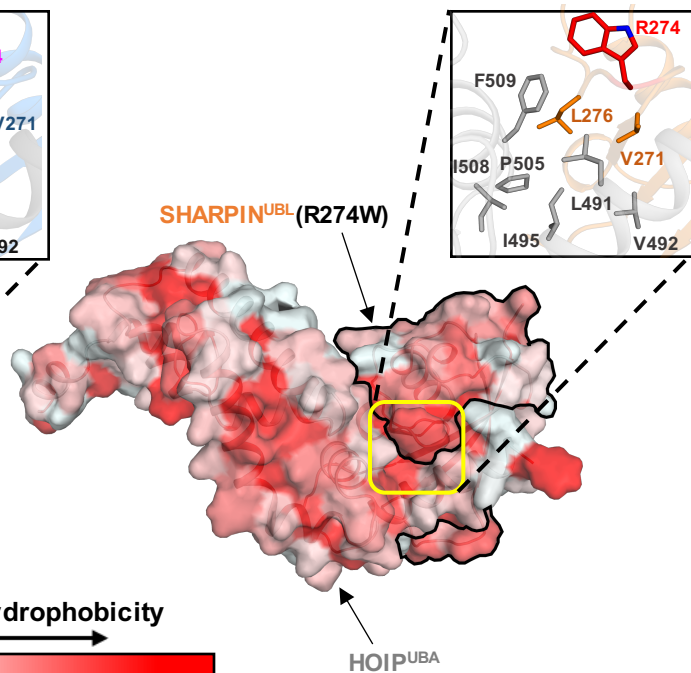

Supplement: Supplementary file 10 — Supplemental Figure8 [file 41398_2021_1680_MOESM10_ESM.pdf]
