# Supplementary material for: A missense variant in SHARPIN mediates Alzheimer’s disease-specific brain damages
Source: Transl Psychiatry. 2021 Nov 16;11:590. doi: 10.1038/s41398-021-01680-5 (PMC8595886; doi:10.1038/s41398-021-01680-5)
Supplement: Supplementary file 8 — Supplemental Figure6 [file 41398_2021_1680_MOESM8_ESM.pdf]

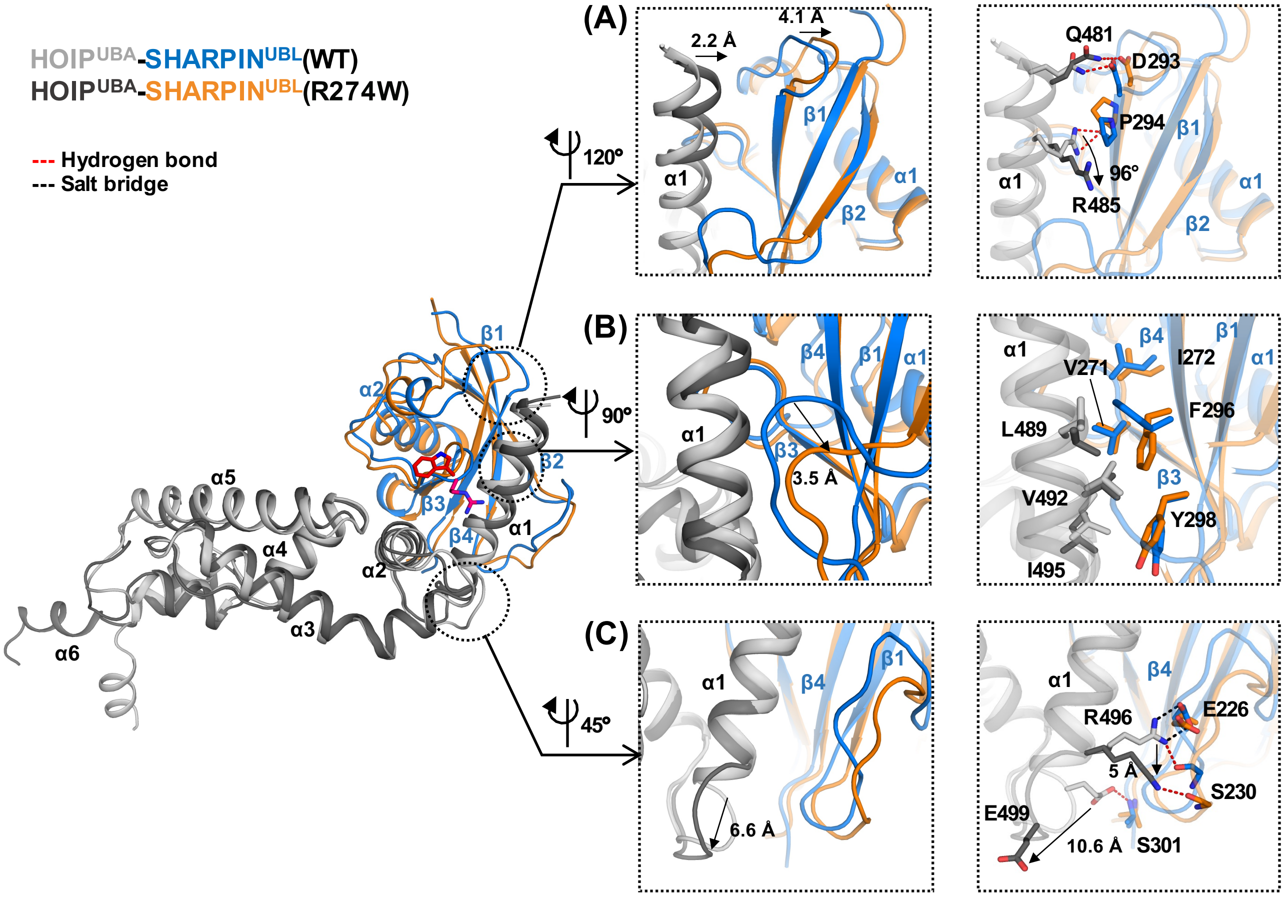

HOIP^UBA-SHARPIN^UBL(WT)
HOIP^UBA-SHARPIN^UBL(R274W)
--- Hydrogen bond
--- Salt bridge
β1
α2
β2
β3
β4
α1
α5
α4
α2
α3
α6
120°
90°
45°
(A)
2.2 Å
4.1 Å
β1
α1
α1
β2
Q481
D293
P294
β1
96°
α1
α1
R485
β2
(B)
β4
β1
α1
α1
β3
3.5 Å
α1
β4
β1
I272
α1
V271
F296
L489
β3
V492
Y298
I495
(C)
α1
β4
β1
6.6 Å
α1
β4
R496
E226
5 Å
S230
E499
10.6 Å
S301
